# Hypoxia and Temperature Regulated Morphogenesis in *Candida albicans*

**DOI:** 10.1371/journal.pgen.1005447

**Published:** 2015-08-14

**Authors:** Prashant R. Desai, Lasse van Wijlick, Dagmar Kurtz, Mateusz Juchimiuk, Joachim F. Ernst

**Affiliations:** 1 Department Biologie, Molekulare Mykologie, Heinrich-Heine-Universität, Düsseldorf, Germany; 2 Manchot Graduate School Molecules of Infection, Heinrich-Heine-Universität, Düsseldorf, Germany; University College Dublin, IRELAND

## Abstract

*Candida albicans* is a common commensal in the human gut but in predisposed patients it can become an important human fungal pathogen. As a commensal, *C*. *albicans* adapts to low-oxygen conditions and represses its hyphal development by the transcription factor Efg1, which under normoxia activates filamentation. The repressive hypoxic but not the normoxic function of Efg1 required its unmodified N-terminus, was prevented by phosphomimetic residues at normoxic phosphorylation sites T179 and T206 and occurred only at temperatures ≤35°C. Genome-wide binding sites for native Efg1 identified 300 hypoxia-specific target genes, which overlapped partially with hypoxic binding sites for Ace2, a known positive regulator of hypoxic filamentation. Transcriptional analyses revealed that *EFG1*, *ACE2* and their identified targets *BCR1* and *BRG1* encode an interconnected regulatory hub, in which Efg1/Bcr1 act as negative and Ace2/Brg1 act as positive regulators of gene expression under hypoxia. In this circuit, the hypoxic function of Ace2 was stimulated by elevated CO_2_ levels. The hyperfilamentous phenotype of *efg1* and *bcr1* mutants depended on Ace2/Brg1 regulators and required increased expression of genes encoding Cek1 MAP kinase and its downstream target Cph1. The intricate temperature-dependent regulatory mechanisms under hypoxia suggest that *C*. *albicans* restricts hyphal morphogenesis in oxygen-poor body niches, possibly to persist as a commensal in the human host.

## Author Summary

*Candida albicans* is an important cause of human disease that occurs if the fungus proliferates strongly on skin surfaces or in several internal organs causing superficial and systemic mycosis. Remarkably, at low cell numbers, *C*. *albicans* is also a normal inhabitant of mucosal surfaces and the gut and it is believed that its transition from the commensal to the virulent, highly proliferative state is a key event that initiates fungal disease. In the gut and other body niches, *C*. *albicans* adapts to an oxygen-poor environment, which downregulates its virulence traits including the ability to form hyphae. We report on a set of four transcription factors in *C*. *albicans* that form an interdependent regulatory circuit, which downregulates filamentation specifically under hypoxia at slightly lowered body temperatures (≤ 35°C). Disturbance of this circuit is expected to initiate the fungal virulence and proliferation in predisposed patients.

## Introduction

*Candida albicans* is a regular fungal inhabitant of the human gastrointestinal tract and the skin [1–3] but in predisposed patients it can also cause life-threatening systemic disease [4]. Systemic candidiasis occurs if resident fungi translocate to the blood and proliferate massively in extraintestinal organs [5,6]. Currently, the requirements for *C*. *albicans* commensalism are being investigated using murine models of colonization, in which fungi are fed orally and monitored during transit and following exit of the gut [7–11]. In all studies, *C*. *albicans* cells growing in the gut lumen were found to propogate in the yeast form and not in the alternative hyphal form. Transciptomal analyses revealed that *C*. *albicans* adapts to conditions in the mouse gut or in internal organs by upregulation of genes related to growth, stress-resistance and cell surface components [12]. Several proteins required for gut colonization were identified by their defective mutant phenotypes [9,10,12]. In contrast, mutants lacking the transcription factor Efg1 or its homologue Efh1 were found to hyperproliferate in the murine gut [7–9], while overproduction of the Efg1-antagonist Wor1 stimulated excessive proliferation [9]. These results suggested that *C*. *albicans* limits its gastrointestinal growth by the repressive transcriptional activity of the Efg1 protein [8]. Gut mucosal damage, deficiencies in immune defenses and defects of the gut probiotic microbiome have been described as essential preconditions to allow translocation and systemic dissemination of *C*. *albicans* originating from the gut [11,13]. Despite this knowledge, the environmental cues and signaling pathways favouring commensal growth of *C*. *albicans* and its transition to the invasion and dissimination states are largely unclear.

Oxygen-poor locations are frequent in the human host and some niches including the gut may be anoxic [14,15], while other tissues including tissue of exposed skin are hypoxic [16,17]. Hypoxia has also been verified in the mouse gastrointestinal tract [18]. *C*. *albicans* adapts to hypoxia by increasing glycolytic and decreasing respiratory metabolism; furthermore, increased expression of genes required for the oxygen-dependent biosynthesis of compounds including ergosterol and unsaturated fatty acids procures maximal use of residual oxygen [19–21]. Under hypoxia, genes required for ergosterol biosynthesis are induced by the transcription factor Upc2 [20,22], while the transcription factors Efg1 and Ace2 both upregulate glycolysis and downregulate oxidative activities [19,23,24]. Efg1 is required for rapid transcriptomal adaptation to hypoxia [25], it controls the regulation of many hypoxic genes and prevents inappropriate hypoxic regulation of normoxic genes [14,19]. Besides their hypoxic metabolic functions, Efg1 and Ace2 also regulate the yeast-to-hypha transition, an important virulence trait of *C*. *albicans*, in an oxygen-dependent manner. Under normoxia, *efg1* mutants are unable to form hyphae indicating that Efg1 acts as an inducer of morphogenesis [26,27]. In contrast, Efg1 represses hyphal growth under hypoxia, which is apparent by hyperfilamentous growth of *efg1* mutants during hypoxic growth on an agar surface [19,23,28] or during embedment in agar [4,29] but not during growth in liquid media. The increased hyperfilamentous phenotype of an *efg1 efh1* double mutant demonstrated further that the Efg1 homolog Efh1 acts synergistically with Efg1 [23]. The function of Efg1 as a hypoxic repressor was strikingly temperature-dependent since *efg1* mutants were hyperfilamentous at temperatures ≤ 35°C, while at 37°C they were unable to form hyphae under both hypoxia and normoxia [19]. In contrast to Efg1, the Ace2 protein was found to be largely dispensable for hyphal morphogenesis under normoxia [30–32] but it was required for filamentation under hypoxia [30,32]. Thus, Efg1 and Ace2 have opposing functions under hypoxia and recent results suggested that Efg1 represses *ACE2* expression [32], as well as expression of Ace2 target genes [32,33] under normoxia.

Both described hypoxic functions of Efg1, i. e. to regulate yeast proliferation of *C*. *albicans in vitro* and in the mouse gut *in vivo*, may be directed by similar if not identical regulatory circuits. In support of this notion, as compared to the wild-type strain, an *efg1* mutant not only was hyperproliferative in the mouse gut [7–9] but it also showed increased extraintestinal dissemination in animals exposed to hypoxia [34] and increased virulence in orally-inoculated mice [35]; in contrast, the virulence of the *efg1* mutant was strongly reduced in the systemic model of bloodstream-infection, i. e. under increased oxygen levels [27,35]. Hypoxia by decreased blood flow in individual gut villi had previously been shown to favor invasion and translocation of *C*. *albicans* across enterocytes [36]. Conceivably, the hypoxic repressor functions of Efg1 are relevant not only at temperatures <37°C, i.e. for fungal colonization of exposed skin tissue but also for translocation across gut epithelia. Here we identify a transcriptional regulatory hub describing the functions of Efg1 under hypoxia that controls the proliferation and morphogenesis of *C*. *albicans* in oxygen-limiting environments.

## Results

### Efg1 hypoxic function requires its unmodified N-terminus

**Fig 1:**
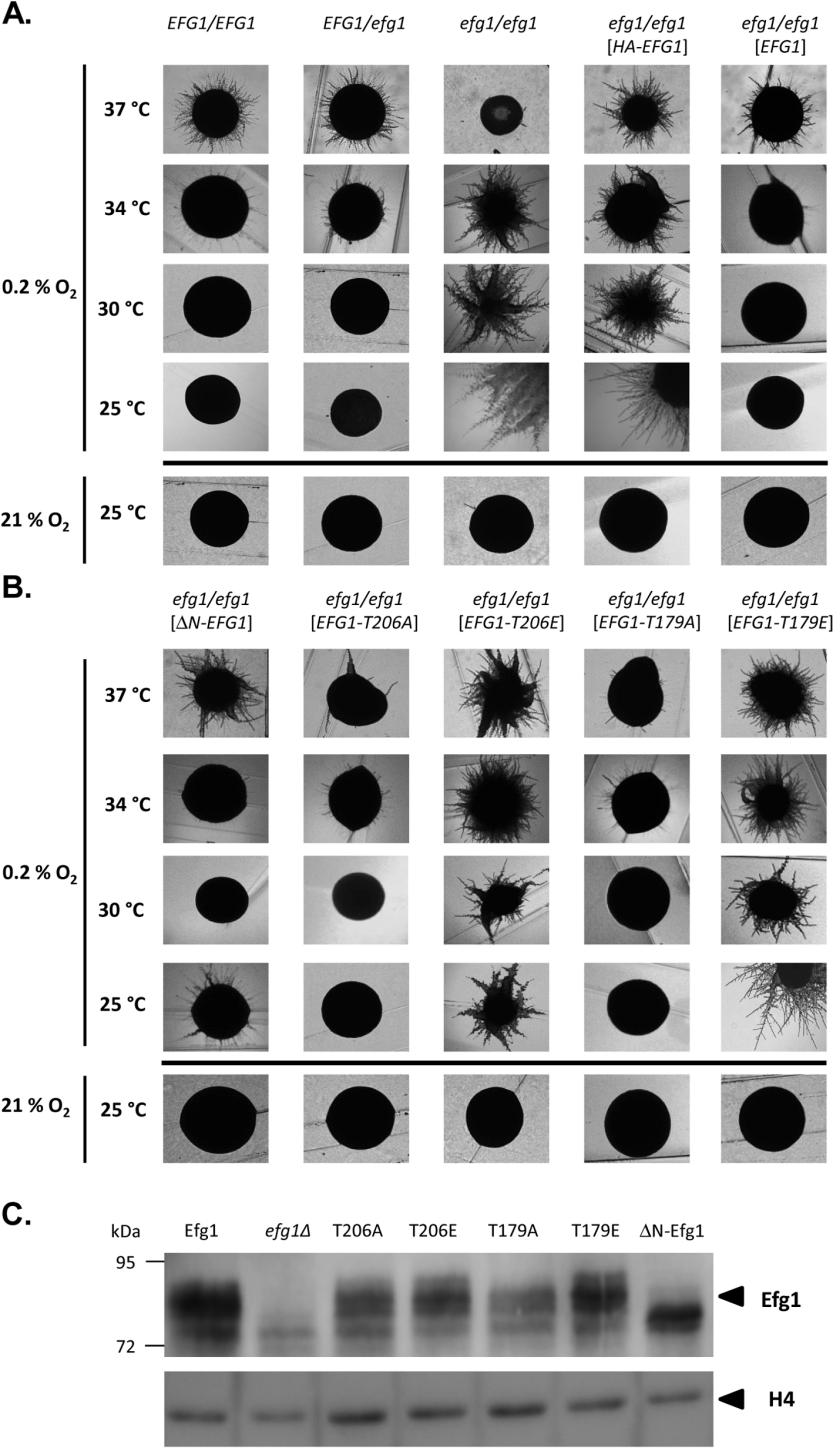
Hypoxia and temperature dependent filamentation regulated by Efg1 variants. (A, B) Colony phenotypes. Strains were grown under hypoxia (0.2% O_2_) on the surface of YPS agar at 37°C (3 d), 34°C (3 d), 30°C (3 d) or 25°C (4 d) and under normoxia (21% O_2_) at 25°C (4 d). (C) Efg1 immunoblot. Strains werμe grown on YPS agar for 60 h at 25°C before cell harvesting by scraping off the colonies. 75 μg of protein in cell extracts were separated by SDS-PAGE (8% acrylamide) and immunoblots were developed using anti-Efg1 antiserum. Levels of histone H4 (loading control) were detected by anti-histone H4 antibodies. Strains tested were CAF2-1 (*EFG1/EFG1*), BCA0901 (*EFG1*/*efg1*), HLC52 (*efg1*/*efg1*), HLCEEFG1 (*efg1*/*efg1* [*HA-EFG1*]), HLCPEFG1 (*efg1*/*efg1* [*EFG1*]), HLCNEFG1 (*efg1*/*efg1* [Δ*N-EFG1*]), HLCEEFG1T206A (*efg1/efg1* [*EFG1-T206A*]), HLCEEFG1T206E (*efg1/efg1* [*EFG1-T206E]*), HLCEEFG1T179A (*efg1/efg1* [*EFG1-T179A*]) and HLCEEFG1T179E (*efg1/efg1* [*EFG1-T79E*]).

Under hypoxia, Efg1 has been described as a temperature-dependent regulator of morphogenesis because it suppresses filamentation during surface growth at temperatures ≤35°C [19], while at 37°C it is required for hyphal growth [19] as under normoxia [26,27]. Properties of the *efg1* mutant were re-confirmed by growth on the surface of YPS agar, which under normoxia does not induce hypha formation in *C*. *albicans* [37]. Under hypoxia (0.2% O_2_), the *efg1* mutant was unable to filament at 37°C, while it showed vigorous hyphal outgrowth at 34, 30 and 25°C; in contrast, under normoxia no filamentation was observed at 25°C (Fig 1A). At 37°C under hypoxia, the defective filamentation of an *efg1* homozygous mutant on YPS agar was fully restored not only by native Efg1 but also by an N-terminally HA-tagged Efg1 variant (Fig 1A) or by an Efg1 variant carrying a N-terminal deletion (Fig 1B), as under normoxia [38]. In contrast, at 25, 30 or 34°C the synthesis of HA-Efg1 did not prevent hyperfilamentation in an *efg1* genetic background, while native unmodified Efg1 had this activity (Fig 1A). The repressing function of authetic Efg1 was slightly reduced by deleting residues 9 to 74 (ΔN-Efg1) since colonies grown at 25°C (but not at 30 or 34°C) showed residual filamentation (Fig 1B). Thus, the morphogenetic repressor function of Efg1 requires its native N-terminus.

The structural requirements for hypoxic Efg1 functions were explored further by single-site mutated variants mutated for residues T206 and T179. T206 fits the consensus sequence for phosphorylation by PKA [39] and T179 is considered as the phosphorylation site of the Cdc28-Hgc1 kinase complex [33]; phosphorylation of both residues is needed for efficient hypha formation under normoxia [33,39]. *EFG1* versions encoding T206A, T206E, T179A and T179E variants were integrated into the genome of an *efg1* mutant and filamentation phenotypes of transformants were examined. All Efg1 variants were produced at similar levels during hypoxic growth (Fig 1C) as under normoxia [38], which was assayed by immunoblotting of cell extracts using a newly generated anti-Efg1 antiserum (S1 Fig). Interestingly, at 25, 30 and 34°C, both non-phosphorylatable variants T206A and T179A effectively repressed filamentation, while Efg1 variants mimicking phosphorylation (T206E and T179E) were unable to act as repressors (Fig 1B). This result suggests that opposite to its normoxic functions, the phosphorylated forms of Efg1 are inactive in hypoxic repression, while the non-phosphorylated forms are active.

Collectively, the results suggest that normoxic and hypoxic functions of Efg1 have different structural requirements. An unmodified native N-terminus of Efg1 and the lack of T206/T179 phosphorylation appear essential for its repressive functions under hypoxia at temperatures slightly below 37°C.

### Genomic binding of native and HA-tagged Efg1 under hypoxia

The above experiments had shown that native but not HA-tagged Efg1 is active for morphogenetic repression under hypoxia at 25°C and 30°C. Next, we sought to identify genes that are hypoxically repressed by Efg1 but not HA-Efg1 under hypoxia. For this purpose genomic binding sites for both Efg1 versions were determined by ChIP chip analyses and compared. Binding of native Efg1 was determined in the Efg1^+^ strain CAF2-1 using anti-Efg1 antibody for immunoprecipitation and the *efg1* mutant HLC52 [27] as the background control strain; binding of HA-Efg1 was established using anti-HA antibody and strain CAF2-1 as the reference control [40]. Cells were grown at 30°C (i. e. a temperature compatible with the hypoxic repressor function of Efg1) in liquid glucose-containing YPD medium. This experimental setup was chosen to focus on hypoxia-regulated targets under clearly defined conditions using uniformly exponentially-growing yeast cells and to exclude other targets, e. g. related to differential filamentation. Furthermore, normoxic targets for HA-Efg1 had been previously determined in identical conditions [40] and provided a useful dataset for comparisons.

**Fig 2:**
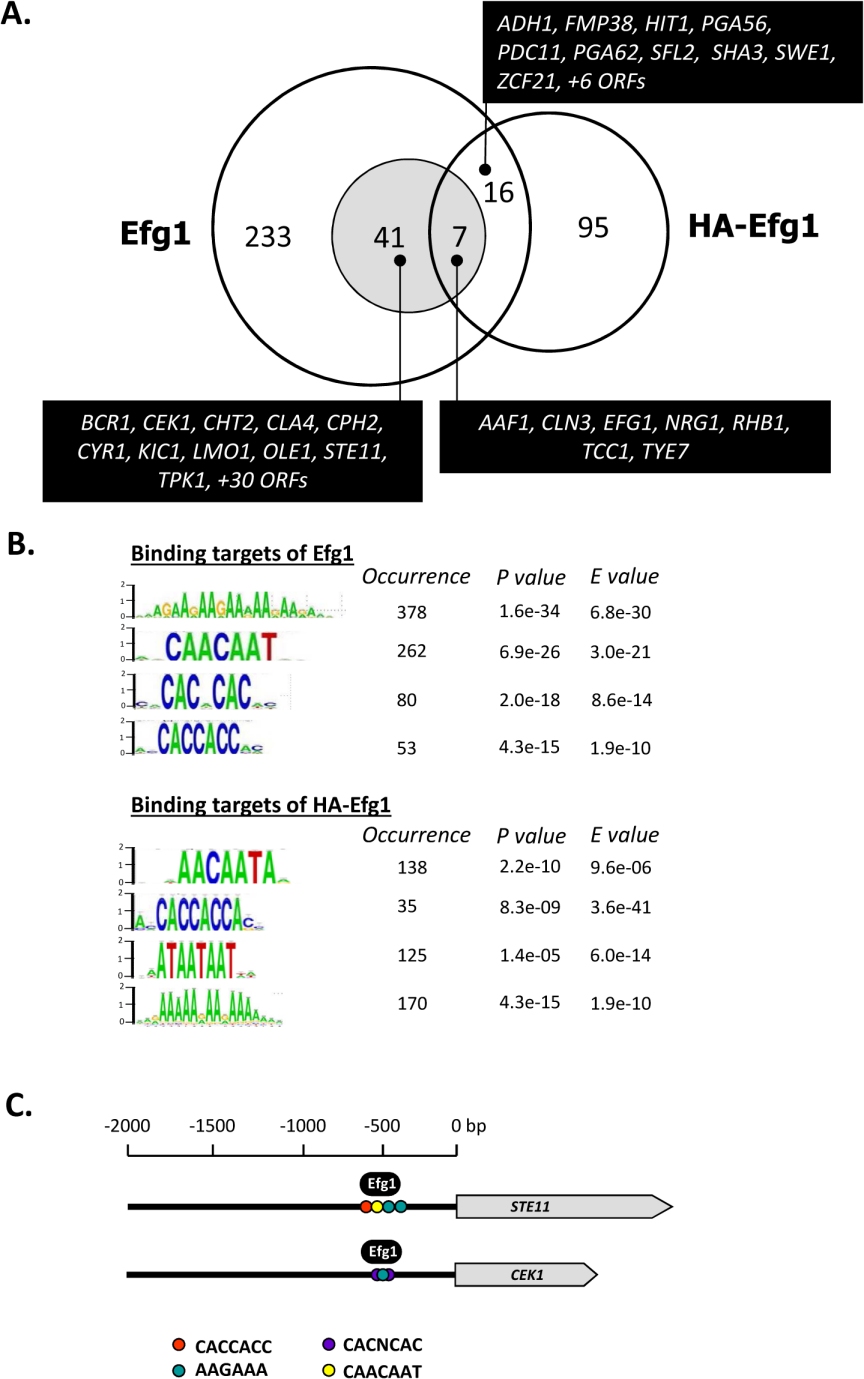
Genomic binding sites for Efg1. (A) Venn diagram listing numbers of genes bound by untagged Efg1 (Efg1) and HA-Efg1 under hypoxia. For Efg1, genomic binding sites were derived from ChIP chip experiments comparing strains CAF2-1 (*EFG1/EFG1*) and HLC52 (*efg1*/*efg1*); for HA-Efg1, strains HLCEEFG1 (*efg1*/*efg1* [*HA-EFG1*]) and DSC11 (*efg1*/*efg1* [*EFG1*]) were compared. The shaded circle encompasses genes in filamentous growth. (B) The program RSAT dyad analysis [43] was used to predict the DNA binding motif of Efg1 and HA-Efg1 from genomic binding regions. Predicted dyads for Efg1 and HA-Efg1 binding sites under hypoxic growth were ranked and the top-ranked sequences are shown with their P-/E-values. (C) Position of Efg1 binding sites in promoter regions. Arrows indicate ORFs of genes *STE11*, *CEK1* and *CPH1* and consensus sequences representing potential Efg1 binding sites in promoter regions are indicated by colored circles. The position of identified Efg1 binding (black oval) in *STE11* and *CEK1* promoters is indicated.

Genomic binding sites for native Efg1 (221 sites corresponding to 300 ORFs) greatly outnumbered those for HA-tagged Efg1 (100 sites corresponding to 118 ORFs) and surprisingly little overlap was found for sites binding both proteins (23 sites); 198 sequences were exclusively bound by native Efg1 under hypoxia (Fig 2A). Little overlap was also detected between targets of HA-Efg1 under hypoxia and normoxia [40] (S2 Fig). Binding sites for both proteins are specified in S1 Table and are available at (http://www.candidagenome.org/download/systematic_results/Desai_2014/). Hypoxic binding occurred exclusively in promoter regions upstream of ORFs, marking these genes as potential regulatory targets (in case of divergently transcribed genes both ORFs were considered as regulatory targets). A significant subset of identified genes has a known or suspected role in hyphal growth of *C*. *albicans* (shaded area in Fig 2A). Genes binding both Efg1 and HA-Efg1 included *EFG1* itself [40], as well as seven genes encoding general morphogenetic regulators comprising *NRG1*, *TCC1* and *TYE7*. Forty-one genes were only bound by native Efg1 but not by HA-Efg1 under hypoxia including *BCR1*, *CEK1*, *CPH2*, *CYR1*, *STE11* and *TPK1*. Consensus sequences in promoters binding Efg1 proteins were calculated using the program RSAT dyad analysis [41] and revealed an enrichment for CA-containing motifs for both Efg1 and HA-Efg1 (Fig 2B) that may represent binding sites. This result suggests that although target promoters differ, untagged and tagged Efg1 proteins bind to identical sequences under hypoxia. Interestingly, the binding sequences resemble CA-containing sequences bound by HA-Efg1 during hyphal induction under normoxia but differ from the major binding site (EGR-box TATGCATA) in normoxically grown, yeast-form cells [40].

**Fig 3:**
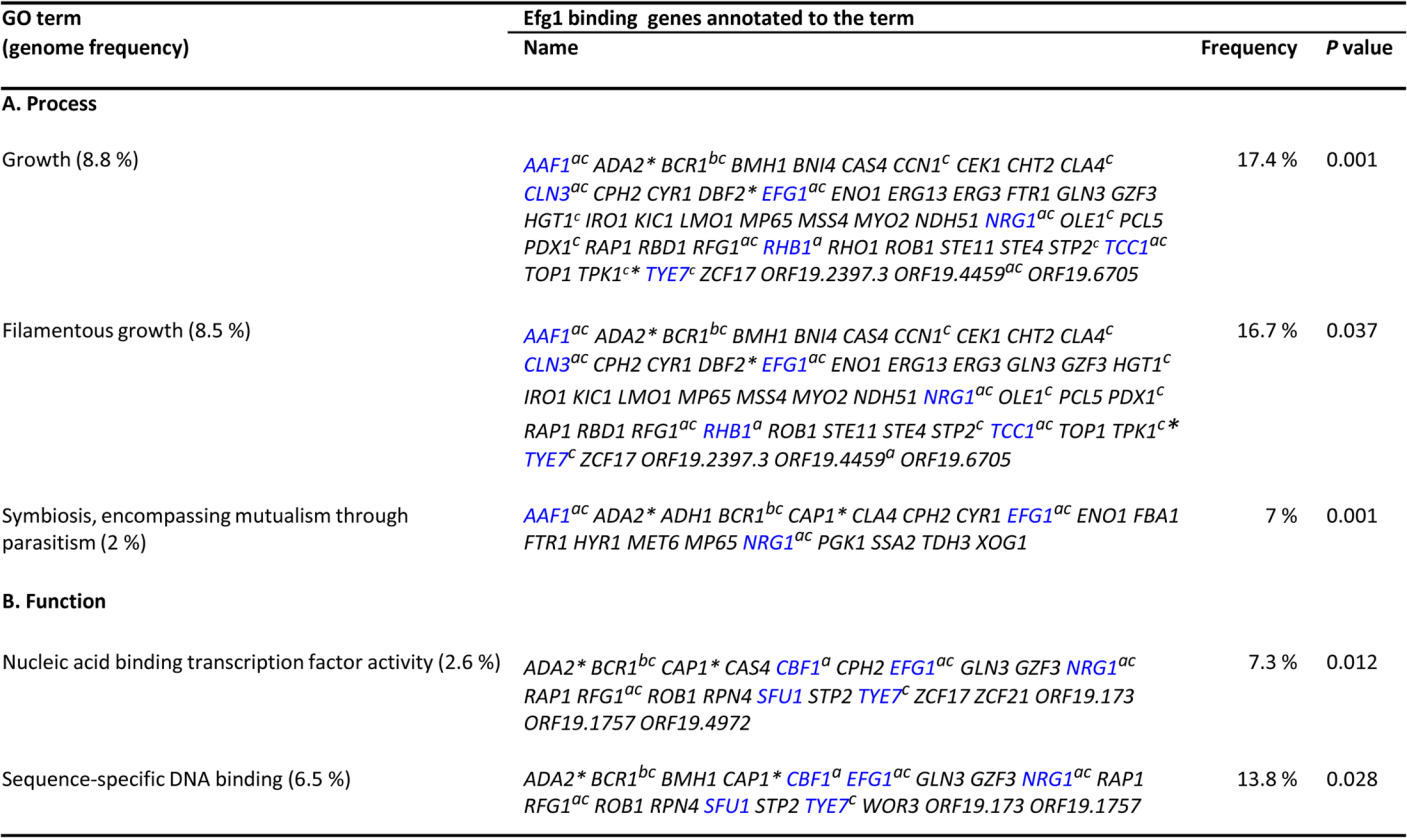
GO categories of genes binding Efg1 under hypoxia. GO terms for Efg1 binding targets were identified in ChIP chip data using the CGD GO Term Finder tool (http://www.candidagenome.org/cgi-bin/GO/goTermFinder); the analysis was conducted in June 2013. Genome frequencies of genes corresponding to GO terms are expressed as percentages (gene number relative to 6,525 genes in the *C*. *albicans* genome; the frequency of genes binding Efg1 that correspond to a specific GO term are expressed relative to the total number of 287 genes binding Efg1). Superscripts of genes indicate known gene functions: a, Efg1 binding under yeast normoxia [40]; b, Efg1 binding in hypha-inducing conditions [40]; c, Efg1 binding in biofilm-inducing conditions [42]; *genes regulated by Efg1 during GI tract colonisation [8]. Blue Lettering indicates common target genes bound by Efg1 and HA-Efg1 under hypoxia. *P* values for overrepresented categories were calculated using a hyper geometric distribution with multiple hypothesis correction according to the GO Term Finder tool. The *P* value cutoff used was 0.05.

Gene ontology (GO) analysis of genes binding native Efg1 under hypoxia revealed an enrichment for genes involved in filamentation and transcription factor activity (Fig 3), as expected from the Venn diagram (Fig 2A). Several of these genes had previously been identified as targets of HA-tagged Efg1 grown under normoxia in liquid [40] or during biofilm formation [42]. Gene ontology assignments for HA-Efg1 are shown in S3 Fig.

### Transcriptional regulation under hypoxia

The above results had indicated a subset of genes bound by native but not HA-tagged Efg1, which are known to be involved in the yeast-to-hypha transition. Efg1 binding in promoter regions of these genes suggested that they are transcriptionally regulated by Efg1, explaining the hypoxic repressor function of Efg1 on hyphal morphogenesis. To verify this notion, transcript levels of selected genes were monitored during the shift from normoxia to hypoxia in Efg1^+^ cells (CAF2-1) and *efg1* mutant cells (HLC52). Genes *STE11*, *CEK1* and *CPH1* encode members of the Cek1 MAP kinase cascade, which is needed for hypha formation of *C*. *albicans* mainly during surface growth [43–45]; Efg1 binding occurs in the promoter region of *STE11* and *CEK1* genes at the CA-type consensus binding sequences (Fig 2C). Transcripts of genes *CYR1* and *TPK1* were also analyzed that encode adenylate cyclase and PKA isoform 1, respectively, which are members of the cAMP-dependent pathway of filamentation [46,47]. In addition, the *KIC1* transcript encoding a presumed regulator of the Ace2 transcription factor [48] was assayed since Ace2 stimulates hypoxic filamentation [30] (see below).

**Fig 4:**
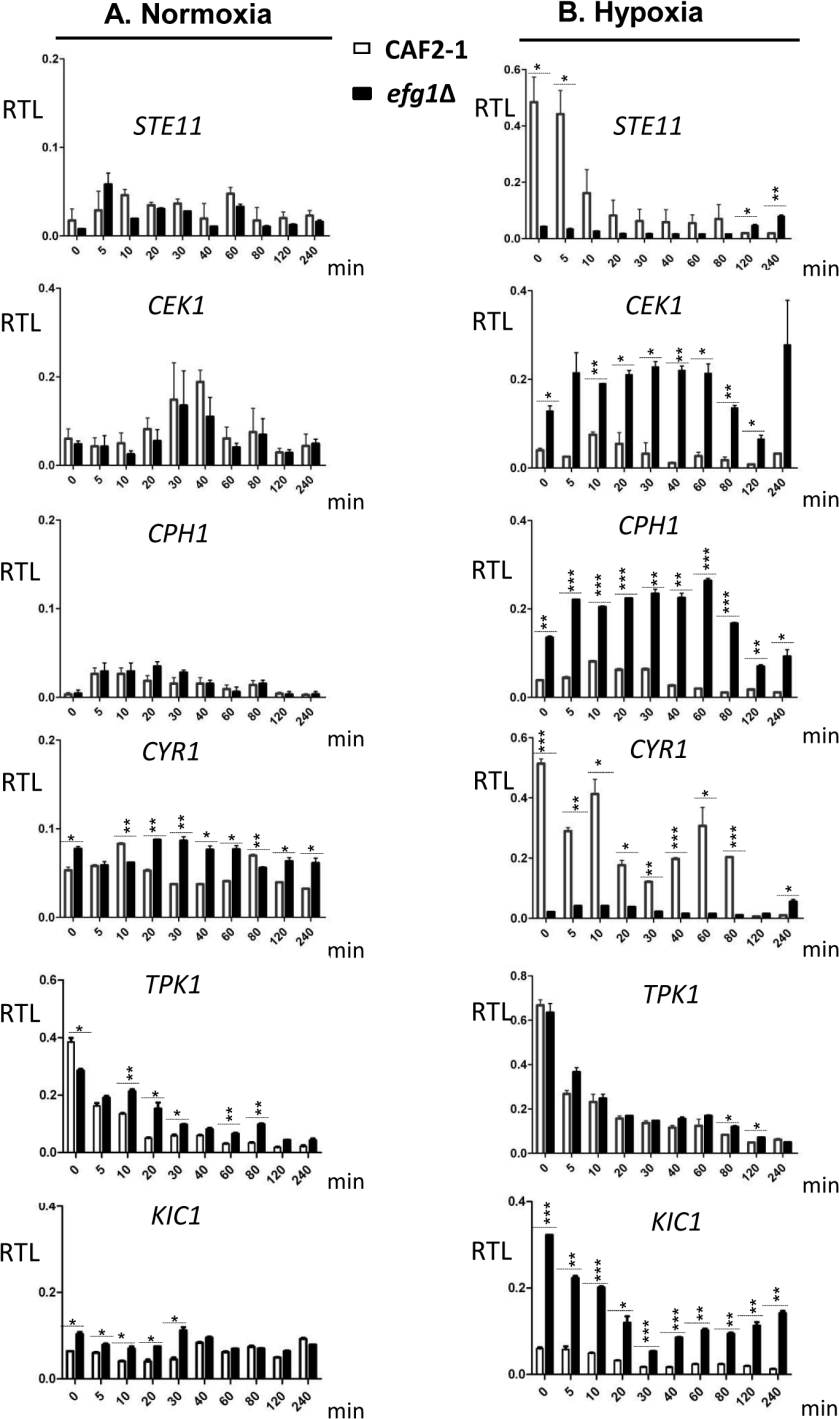
Transcriptional regulation of Efg1 target genes under normoxia and hypoxia. Strains were precultured under normoxia at 30°C in YPD medium and used for inoculation of 200 ml YPD cultures for normoxia, or under 0.2% O_2_ for hypoxia. Cultures were incubated at 30°C under normoxia or hypoxia, and at the indicated times 20 ml of culture was withdrawn and used for preparation of total RNA. At each time point, two biological replicates and three technical replicates were assayed by qPCR using gene-specific primers. mRNA levels are expressed as means ± SEM of transcript levels relative to the *ACT1* transcript (RTL), for normoxia (A) and hypoxia (B) grown cultures. A two-tailed, unpaired *t* test comparing the RTL values of control strain CAF2-1 and *efg1* mutant HLC52 was used to determine the statistical relevance. *, *P* < 0.05; **, *P* < 0.01; ***, *P* < 0.001.

In the control strain, transcripts for the Cek1 MAP kinase, its downstream transcription factor Cph1 and for the Kic1 protein were present at low levels but increased temporarily at 10–20 min following the hypoxic shift (Fig 4). Remarkably, in *efg1* mutant cells, these transcripts were strongly upregulated suggesting hypoxic repression by Efg1. A completely different pattern of regulation was detected for the *STE11* gene encoding a kinase upstream of Cek1, as well as for the *CYR1* adenylate cyclase gene. Transcript levels for both of these genes decreased strongly during the hypoxic shift in the Efg1^+^ strain and were significantly downregulated in the *efg1* mutant. Thus, the regulation of both *STE11* and *CYR1* did not fit the pattern of an Efg1-repressed but rather of an Efg1-induced gene; in addition, expression of both genes was down- and not upregulated during the course of hypoxic exposure. The *TPK1* transcript also was downregulated under hypoxia but the absence of Efg1 did not affect its levels. Collectively, these results suggest that under hypoxia Efg1 downregulates *CEK1*, *CPH1* and *KIC1* transcript levels to suppress filamentation, which becomes evident by the *efg1* mutant phenotype (Fig 1). Efg1 binding to *STE11*, *CYR1* and *TPK1* promoters may have other functions that are not directly related to repression of hypoxic filamentation.

### Efg1 represses Cek1-dependent filamentation under hypoxia

**Fig 5:**
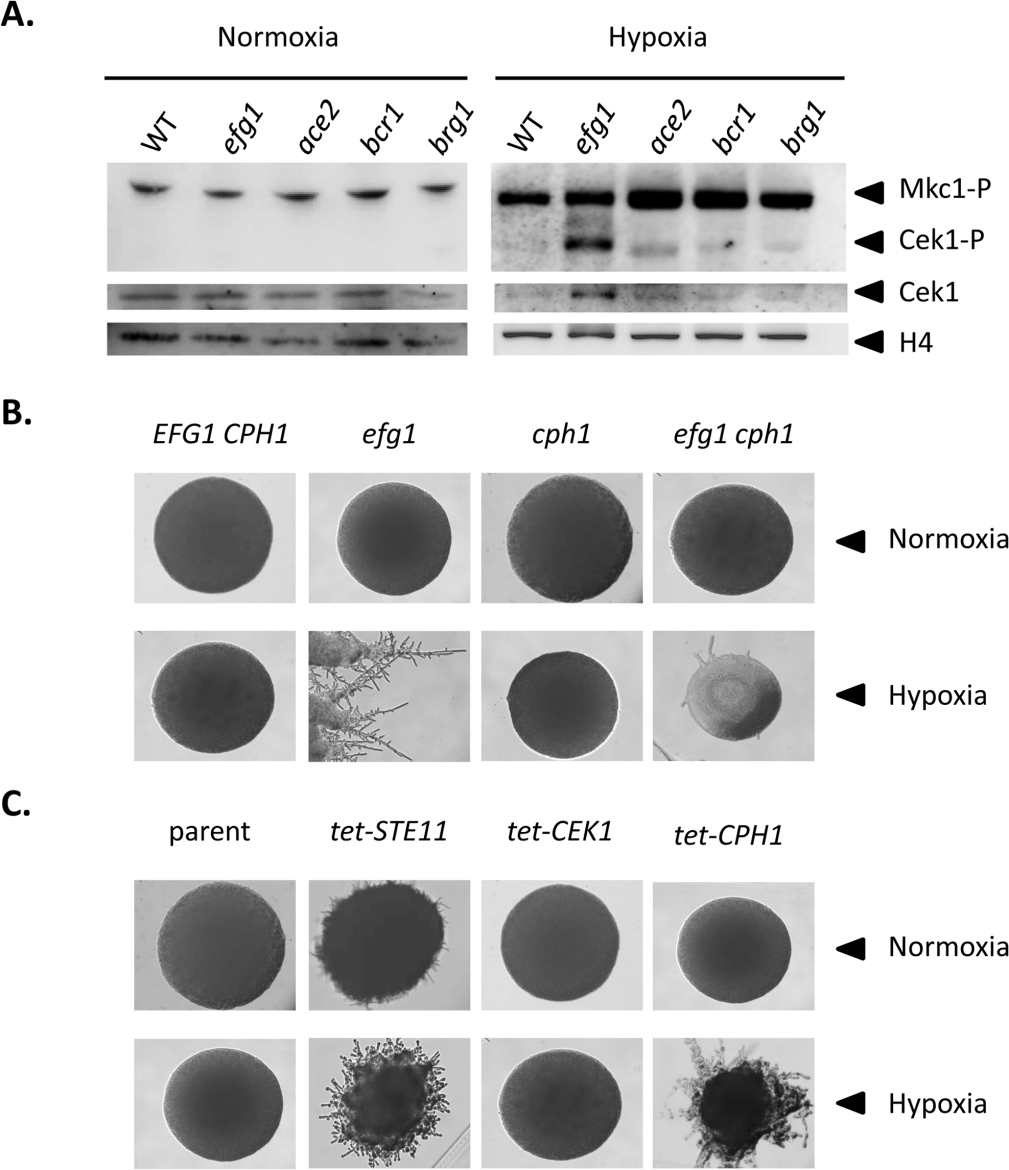
Hypoxic filamentation is activated by the Cek1 pathway. (A) Levels of MAP kinases in *C*. *albicans* mutants. *C*. *albicans* strains CAF2-1 (WT), HLC52 (*efg1*), MK106 (*ace2*), CJN702 (*bcr1*) and TF022 (*brg1*) were plated on YPS agar plates and incubated for 60 h at 25°C under normoxia or hypoxia (0.2% O_2_). Colonies were scraped off the agar and cell extracts were used for immunoblotting. Anti-phospho-p44/42 antibody was used to detect the phosphorylated forms of MAP kinases Mkc1p and Cek1p. Total Cek1 (Cek1) and histone H4 levels (loading control) were detected by anti-Cek1 and anti histone H4 antibodies, respectively. (B, C) Phenotypes of *C*. *albicans* strains with altered expression of Efg1 target genes. Filamentation phenotypes were monitored following growth on YPS agar at 25°C under normoxia or hypoxia (0.2% O_2_) for 3 d. (B) Mutant phenoypes. Comparisons of control strain CAF2-1 (*EFG1 CPH1*) and homozygous mutants HLC52 (*efg1*), JKC19 (*cph1*), HLC54 (*efg1 cph1*). (C) Overexpression phenotypes. Transformants expressing genes *STE11*, *CEK1* and *CPH1* under control of a tetracycline inducible promoter were grown on YPS medium containing 3 μg/ml anhydrotetracycline. Strains included CDC2907 (parent control), CECSTE11 (*tet-STE11*), CECCEK1 (*tet-CEK1*) and CDCCPH1 (*tet-CPH1*).

To verify the transcriptional data we analyzed levels of the Cek1 MAPK protein and of its phosphorylated form by immunoblotting in wild-type and mutant strains grown under hypoxia and normoxia. Under hypoxia, the total amount of Cek1 and of its phosphorylated form (Cek1-P) was strongly increased in the *efg1* mutant as compared to the wild-type strain, while under normoxia Cek1 levels were unaltered in the mutant and Cek1-P was not detected (Fig 5A). This result matches the observed increase in the *CEK1* transcript level in the *efg1* mutant (Fig 4B). Activation of MAP kinase activity was specific for Cek1 since the Mkc1 phosphorylation status was unaffected by the presence of Efg1 (Fig 5A).

To test if during hypoxic surface growth, excessive filamentation by the Cek1-Cph1 pathway is suppressed by the Efg1 protein we examined filamentation phenotypes under hypoxia in strains lacking or overproducing potential regulator proteins. We observed that a *cph1* mutant and colonies of an *efg1 cph1* double mutant did not form hyphae, unlike the hyperfilamentous *efg1* mutant (Fig 5B). In addition, overexpression of *STE11* and *CPH1* genes encoding members of the Cek1 signaling pathway by an anhydrotetracyclin-inducible promoter stimulated filamentation in the wild-type genetic background (Fig 5C). In this experiment, the failure of overexpressed *CEK1* to induce filamentation may reflect low activity of non-phosphorylated Cek1 in the absence of activation by an upstream kinase. Collectively, the results provide strong evidence that Efg1 represses hypha formation of *C*. *albicans* under hypoxia by repressing the biosynthesis and activity of the Cek1 MAP kinase pathway.

### Intersection of Ace2 and Efg1 regulatory circuits

Ace2 is a transcription factor that under hypoxia, unlike Efg1 at lower temperatures, is required for filamentation [30]. Efg1 represses the transcription of *ACE2* and of Ace2-dependent genes and binds to the *ACE2* promoter under normoxia [31, 32]. On the other hand, Ace2 and Efg1 have similar functions to stimulate glycolysis and to repress oxidative metabolism [23, 30]. These results suggested that Ace2 and Efg1 regulatory circuits overlap to jointly control filamentation of *C*. *albicans* under hypoxia. To verify this notion we compared genomic binding sites of Ace2 and Efg1 in cells grown under hypoxia. Strain CLvW004 (*ACE2-HA/ace2*) was constructed, which synthesizes the Ace2 protein with an added C-terminal triple HA-tag. This strain did not show any of the known *ace2* mutant phenotypes for antimycin A resistance, wrinkled colony growth [24,30] and sensitivity to the Pmt1 *O*-mannosylation inhibitor [49] indicating that the Ace2-HA fusion protein is functional (S4 Fig). For the identification of hypoxic Ace2 genomic binding sites, based on preliminary results, a broad ChIP chip screening strategy was chosen to identify all hypoxic targets including those targets requiring the presence of CO_2_. For this purpose genomic binding sites for Ace2-HA in strain CLvW004 were determined following growth in 0.2% O_2_/ 6% CO_2_ and related to results of strain BWP17 synthesizing unmodified Ace2 for background correction; in parallel, normoxic binding sites were determined. Binding sites for Ace2 are listed in S2 and S3 Tables and deposited at http://www.candidagenome.org/download/systematic_results/Desai_2014/.

**Fig 6:**
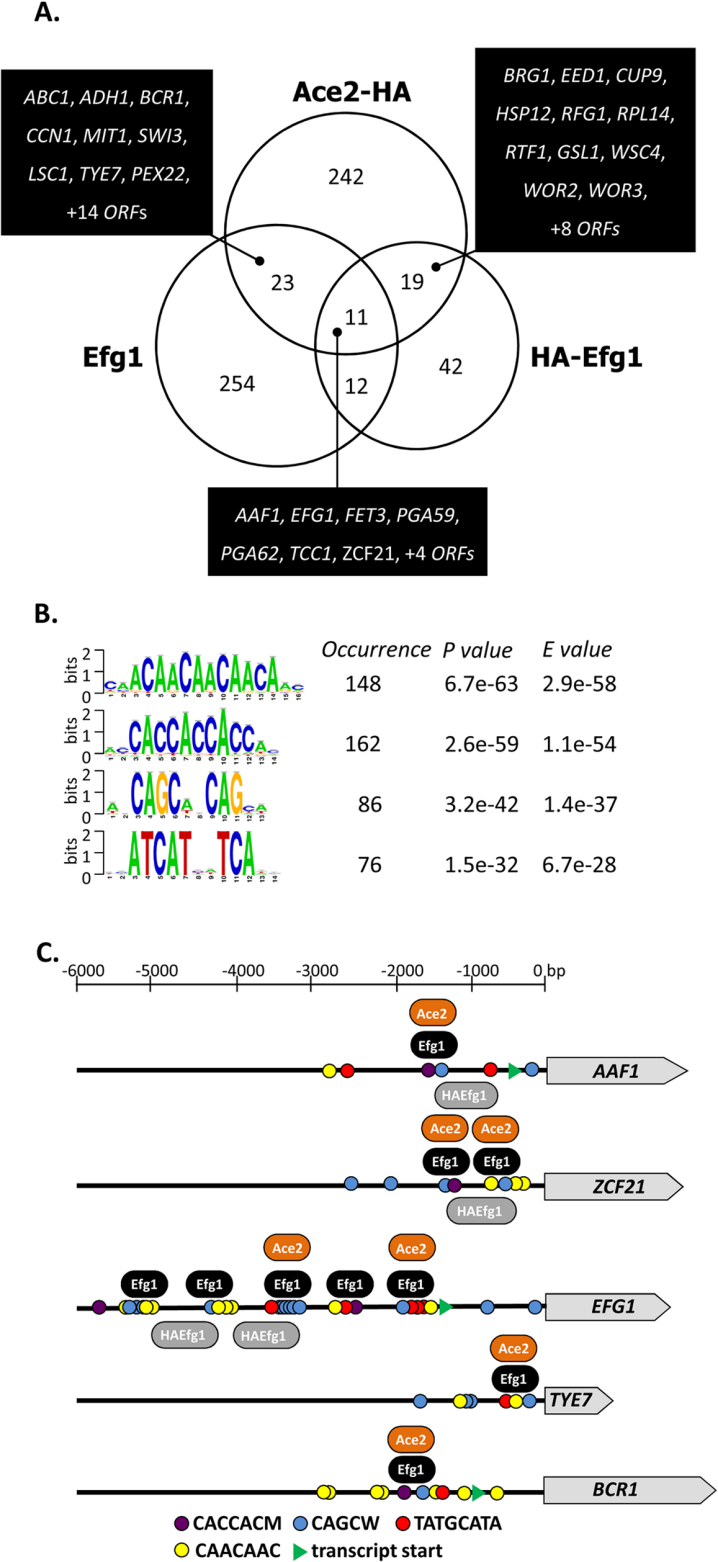
Genomic binding sites for Ace2. (A) Venn diagram showing numbers of genes bound by untagged Efg1 (Efg1), HA-Efg1 and Ace2-HA under hypoxia. For Efg1, genomic binding sites were derived from ChIP chip experiments comparing strains CAF2-1 (*EFG1/EFG1*) and HLC52 (*efg1*/*efg1*), for Ace2-HA, strains CLvW004 (*ACE2-HA/ace2*) and BWP17 were compared. A set of 34 genes is common for Efg1 and Ace2-HA. (B) The program RSAT dyad-analysis [41] was used to predict the DNA binding motif of Ace2-HA from genomic binding regions. Predicted dyads for Ace2-HA binding sites under hypoxic growth were ranked and the top-ranked sequences are shown with their respective *P*- and *E*-values. (C) Position of Efg1 and Ace2-HA binding sites in promoter regions of target genes. Arrows indicate ORFs of genes *AAF1*, *TYE7*, *ZCF21*, *EFG1* and *BCR1*. Consensus sequences representing potential Efg1 and Ace2-HA binding sites in promoter regions are indicated by colored circles. The position of common identified sequences for Efg1 (black oval) and Ace2-HA (orange oval) binding in *AAF1*, *TYE7*, *ZCF21*, *EFG1* and *BCR1* promoters is indicated.

296 significant genomic Ace2 binding sites were identified in *C*. *albicans* promoter regions, while no binding occurred within ORFs (Fig 6A). The majority of binding sites (>80%) was identical in cells grown under hypoxia or normoxia (S5 Fig). Analysis with the RSAT program dyad analysis [41] revealed potential consensus binding sequences CAACAA, CACCAC, CAGCW and ATCAT for Ace2 (Fig 6B). The sequence CAGCW is similar to the CCAGC motif deduced from transcriptomal analyses of Ace2 [30] and matches the binding sequence of *S*. *cerevisiae* Ace2 [50]. Interestingly, the CAACAA and CACCAC motifs had also been observed as potential binding sites for native and tagged Efg1 (Fig 2B) suggesting that these sequences are targeted by both Ace2 and Efg1. Genomic positions for both proteins correspond to binding motifs, mostly to the CACCAC sequence, in a selected group of promoters (Fig 6C).

**Fig 7:**
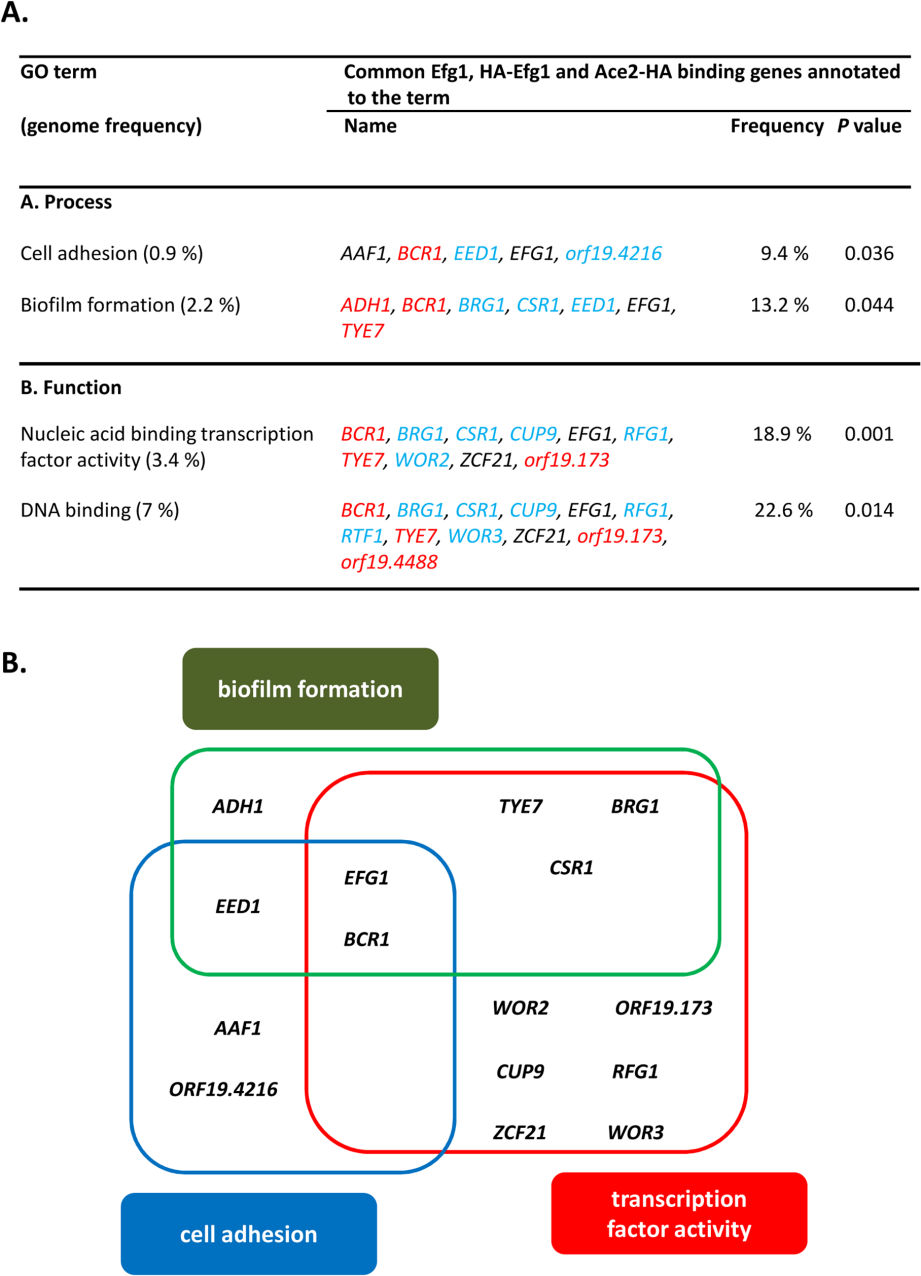
GO categories of common untagged Efg1 (Efg1), HA-Efg1 and Ace2-HA target genes under hypoxia. (A) GO terms for target genes identified by ChIP-chip analysis that are common for untagged Efg1, HA-Efg1 and Ace2-HA under hypoxia using the CGD GO Term Finder tool (http://www.candidagenome.org/cgi-bin/GO/goTermFinder); the analysis was conducted in May 2014. Genome frequencies of genes corresponding to GO terms are expressed as percentages (gene number relative to 6,525 genes in the *C*. *albicans* genome; the frequency of genes that correspond to a specific GO term are expressed relative to the total number of 53 genes with either a binding region of untagged Efg1 or HA-Efg1 and Ace2-HA present in their 5’-UTR). *P* values for overrepresented categories were calculated using a hyper geometric distribution with multiple hypothesis correction according to the GO Term Finder tool. The *P* value cutoff used was 0.05. Colors indicate genes binding only untagged Efg1 (red), only HA-tagged Efg1 (blue) or both Efg1 versions (black). (B) Scheme depicting overlap between GO categories for common Efg1 and Ace2 target genes.

53 promoters bound both Ace2-HA and Efg1 and/or HA-Efg1 under hypoxia (Fig 7A). Gene ontology analysis of the corresponding genes revealed their preferential function as transcription factors to regulate processes of cell adhesion, biofilm formation and morphogenesis (Fig 7B). The transcription factors Brg1 [51] and Bcr1 [52] are known to regulate morphogenesis under normoxia but they also appear to function under hypoxia because they are under joint control of both Ace2 and (HA-) Efg1 in this environment (Fig 7B). Other common hypoxic Ace2/Efg1 target genes encode regulators with more specific functions including Aaf1 [53], Adh1 [54], Eed1 [55], Tye7 [56], Rfg1 [57], Wor2 [58], Wor3 [59] and Zcf21 [60]. In control experiments, binding targets were verified by qPCR following ChIP demonstrating strong enrichment of binding sites for Efg1, HA-Efg1 or Ace2-HA in a selected group of target promoters (S6 Fig). By this sensitive method, binding of HA-Efg1 (in addition to Efg1) was also detected at the *BCR1* promoter; furthermore, these data demonstrated the specificity of antibodies used for immunoprecipitation since anti-Efg1 antibody precipitated both Efg1 and HA-Efg1, while anti-HA antibody was specific for HA-Efg1 and Ace2-HA (S6 Fig).

In addition, 242 genes were identified that only bound Ace2-HA but not (HA-)Efg1. This group was enriched for genes involved in glycolysis and oxidative metabolism (e. g. *PFK2*, *ACO1*, *LSC1*) confirming previous transcriptomal analyses [30]. Interestingly, genes involved in mitochondrial translation (*NAM2*, *ORF19*.*4929*, *ORF19*.*4705*, *EAF7*, *PIM1*) were also identified among Ace2 targets. The promoter of the *SCH9* gene encoding a kinase repressing hypha formation under hypoxia if CO_2_ is present [37], was also identified as a hypoxic binding target of Ace2. Collectively, the group of “Ace2-only” genes appears to regulate metabolism and growth but also contains some genes for some relevant morphogenetic regulators including *FLO8* [61,62], *CAS5* [63], *SFL1* [64,65] and *WOR1* [58]. Conceivably, under hypoxia activation of *FLO8*, which is known to be required for CO_2_ sensing [62], may be mediated by Ace2 (see below).

### Efg1-Ace2 and its targets form an interdependent regulatory hub under hypoxia

**Fig 8:**
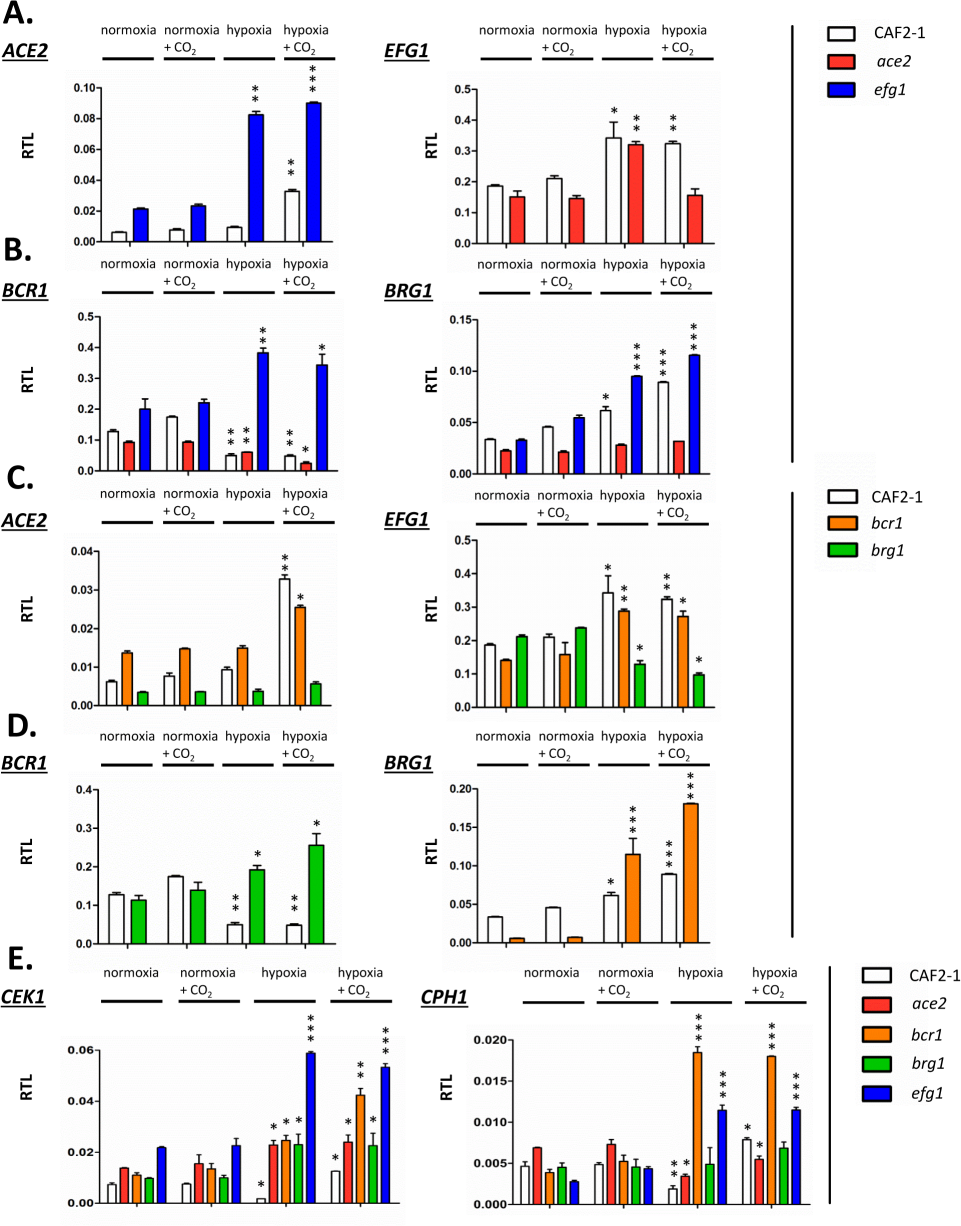
Transcriptional regulation of Efg1 and Ace2 target genes under hypoxia. Strains CAF2-1 (control), the *ace2* mutant MK106 and the *efg1* mutant HLC52 were precultured under normoxia at 30°C in YPD medium and used for inoculation of 100 ml YPD cultures. Cultures were incubated at 30°C under normoxia, under normoxia with addition of CO_2_ (6%), under hypoxia (0.2% O_2_) or under hypoxia with addition of CO_2_ (0.2% O_2_, 6% CO_2_) until OD_600_ = 0.5 and total RNA was then isolated. Relative transcript levels were determined using *ACT1* transcript as the reference, as described in Fig 4. (A) Relative transcript levels (RTL) for the *ACE2* transcript in strains CAF2-1 and HLC52 (*efg1*) and for the *EFG1* transcript in strains CAF2-1 and MK106 (*ace2*). (B to D) Relative transcript levels for the indicated transcripts in strains CAF2-1 (control), HLC52 (*efg1*), MK106 (*ace2*), CJN702 (*bcr1*) and strain TF022 (*brg1*). (E) Relative levels of *CEK1* and *CPH1* transcripts. Error bars represent standard deviation of the means. A two-tailed, unpaired t test comparing the cycle threshold values of samples grown in hypoxic and normoxic conditions for each mutant was used to determine the statistical relevance: *, *P* < 0.05; **, *P* < 0.01; ***, *P* < 0.001.

Joint binding of Efg1 and Ace2 to target promoters under hypoxia suggested that both proteins regulate the respective genes on the transcriptional level. To clarify a specific role of hypoxia on gene regulation the transcript levels of selected Ace2-Efg1 target genes were determined under hypoxia and normoxia. Wild-type cells, as well as *ace2* and *efg1* mutant cells, were grown under normoxia and hypoxia (0.2% O_2_) both in the absence or presence of CO_2_ (6% CO_2_); hypoxia in combination with elevated CO_2_ levels was tested because previous results had suggested that this environment triggers specific patterns of gene expression [37]. Transcript levels were determined for Ace2-Efg1 target genes under hypoxia and normoxia (Fig 8).

First, mutual regulation of *EFG1* and *ACE2* was examined. In the wild-type strain, the *ACE2* transcript was strongly upregulated under hypoxia but only in the presence of CO_2_; upregulation did not require CO_2_ in the *efg1* mutant (Fig 8A) suggesting that Efg1 strongly represses *ACE2* under hypoxia also in the absence of CO_2_. The *EFG1* transcript level was upregulated about twofold under hypoxia in the wild-type strain; this occurred even in the *ace2* mutant in the absence but not in the presence of CO_2_. This mutual regulatory pattern of both genes indicated that under hypoxia, Efg1 acts as a transcriptional repressor independently of CO_2_, while Ace2 requires CO_2_ for its induction activity.

In a similar manner we analysed the hypoxic expression of two Ace2-Efg1 target genes encoding key morphogenetic regulators during biofilm formation under normoxia (*BCR1*, *BRG1*) (Fig 8B). Bcr1 is a positive regulator of biofilm formation, cell surface composition and filamentation [52,66,67], while Brg1 (Gat2) promotes hypha-specific gene expression during hyphal elongation [51,68,69] and promotes *ACE2* expression under normoxia [32]. The *BCR1* transcript was downregulated in the wild-type strain but upregulated in the *efg1* mutant under normoxia but more strongly under hypoxia revealing Efg1 as a strong hypoxic repressor of *BCR1*. In the *ace2* mutant the *BCR1* transcript was downregulated more strongly in the presence than in the absence of CO_2_ suggesting that Ace2 upregulates *BCR1* in this environment, counteracting Efg1-mediated repression. In contrast to *BCR1* regulation, *BRG1* expression was upregulated in the wild-type strain under hypoxia and this upregulation was even enhanced in an *efg1* mutant but reduced in the *ace2* mutant. Thus, although *BCR1* and *BRG1* genes are regulated differently under hypoxia, Efg1 and Ace2 regulate these genes similarly, with Efg1 acting as repressor and Ace2 as an inducer of gene expression. Other genes encoding relevant transcription factors, including Tye7 [56], Aaf1 [53] and Zcf21 [60,70], were also controlled by Efg1/Ace2 (S7 Fig); these proteins regulate glycolysis, biofilm formation and/or commensalism of *C*. *albicans*.

In a previous report Bcr1 and Brg1 had been shown to bind to the *EFG1* promoter [42] suggesting feedback regulation between *EFG1-ACE2* and *BCR1/BRG1* genes under normoxia. To clarify the regulation under hypoxia *ACE2* and *EFG1* transcript levels were determined in *bcr1* or *brg1* mutants. The *ACE2* transcript was strongly downregulated in the *brg1* mutant in all conditions and largely increased in the *bcr1* mutant (not further increasing the already elevated level under hypoxia/CO_2_) (Fig 8C). Thus, Brg1 activates and Bcr1 represses *ACE2* transcript levels. Brg1 also functions as an activator of the *EFG1* transcript under hypoxia, which did not increase in the *brg1* mutant in this condition.

On the other hand, gene products of *BCR1* and *BRG1* also mutually acted as negative regulators since the hypoxic downregulation of the *BCR1* transcript did not occur in a *brg1* mutant (showing even transcript upregulation) and the *BRG1* transcript was upregulated in the *bcr1* mutant under hypoxia (Fig 8D). Under normoxia, however, the *BRG1* transcript was strongly reduced in the *bcr1* mutant indicating that Bcr1 is a normoxic inducer but a hypoxic repressor for *BRG1*. Collectively, the results indicate that *EFG1*, *ACE2*, *BCR1* and *EFG1* genes form an interconnected regulatory hub, in which each participant regulates expression of the co-regulators. The transcriptional output of this unit is specific for hypoxia and is influenced significantly by CO_2_ levels.

### The Efg1-Ace2 regulatory hub regulates hyphal morphogenesis via the Cek1 pathway under hypoxia

The above transcript analyses had revealed that both *CEK1* and *CPH1* genes are repressed by Efg1 under hypoxia (Fig 4). Because Efg1 is part of an interconnected regulatory hub we re-examined the hypoxic/normoxic ratios of both transcripts in the respective mutant backgrounds (Fig 8E). In the wild-type strain, *CEK1* and *CPH1* transcripts were lowered in a hypoxic atmosphere without CO_2_ but reached normoxic levels in the presence of CO_2_ (Fig 8E). The repressive effect of Efg1 on both genes in this environment was clearly evident by strongly increased transcript levels in the *efg1* mutant. Under normoxia the Efg1 co-regulators Ace2, Bcr1 and Brg1 did not greatly influence *CEK1* or *CPH1* transcript levels. However, under hypoxia these regulators all repressed the *CEK1* transcript, while the *CPH1* transcript was downregulated only by Bcr1 (and Efg1). Consistently, protein levels of Cek1 and its phosphorylated form Cek1-P was upregulated under hypoxia (Fig 5A). Collectively, these results confirm the conclusion that Efg1 and its co-regulators control the Cek1 MAP kinase pathway.

**Fig 9:**
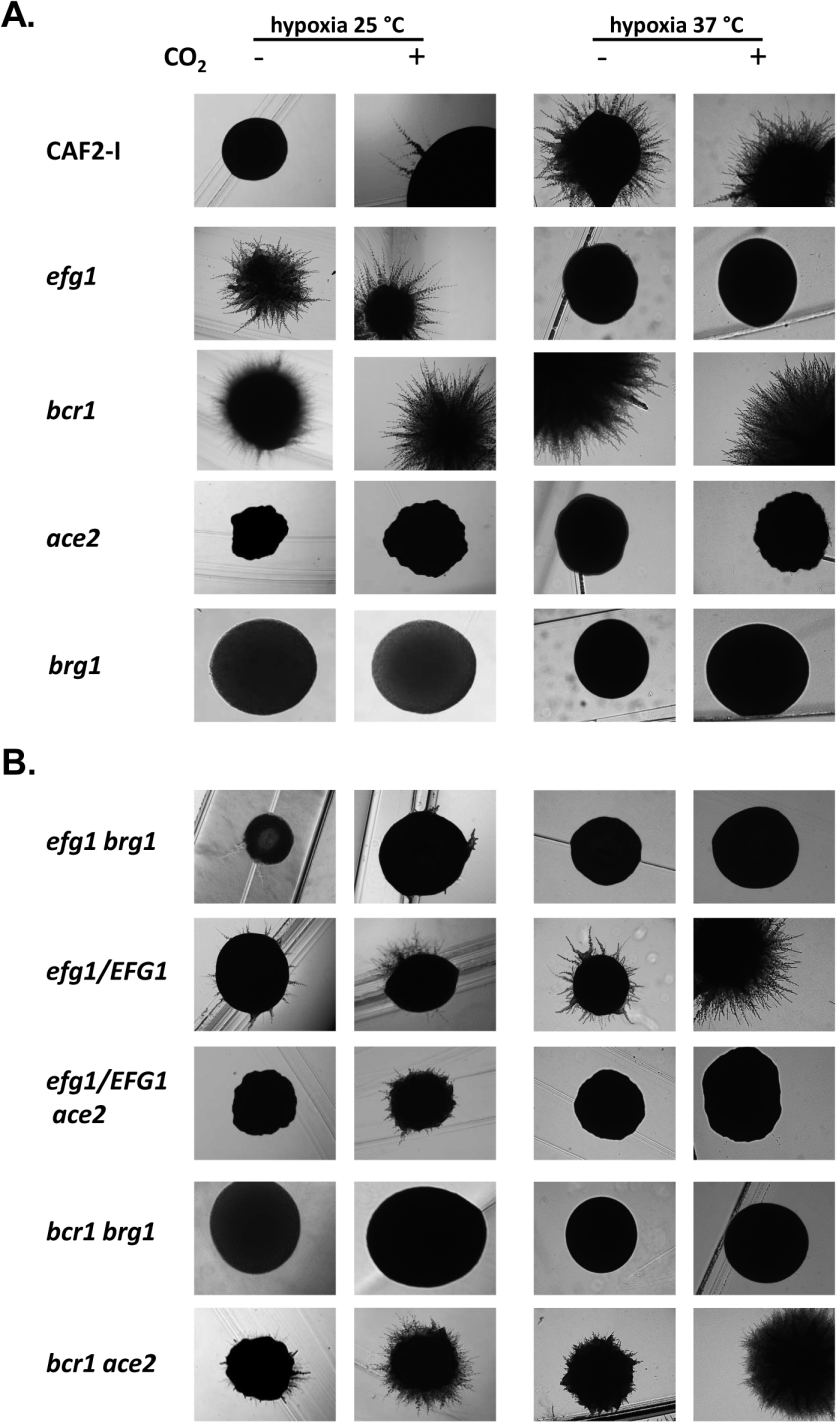
Hypoxic phenotypes of mutants lacking hypoxic regulators. The strains were grown under hypoxia (0.2% O_2_) or hypoxia with 6% CO_2_ on YPS agar for 4 d at 25°C or at 37°C for 3 d. (A) Strains included CAF2-1 (control), homozygous single mutants HLC52 (*efg1*), CJN702 (*bcr1*), MK106 (*ace2*) and TF022 (*brg1*). (B) Double knockout strains PDEB4 (*efg1 brg1*), PDBB4 (*bcr1 brg1*) and CLvW024 (*bcr1 ace2)* were tested. *ACE2* could not be disrupted in an *efg1* mutant background; therefore, the heterozygous mutant strain CLvW047 (*efg1/EFG1*, *ace2*/*ace2*) was constructed and its phenotype was compared to the *efg1/EFG1* strain DSC11.

To examine if and how members of the Efg1-Ace2 regulatory hub influence hyphal morphogenesis the colony phenotypes of control and mutant strains were recorded. Cells were grown on YPS agar under hypoxia or normoxia, in the absence or presence of 6% CO_2_ and at 25°C or 37°C. Under hypoxia, the control strain CAF2-1 showed no or sparse filamentation at 25°C but strong hypha formation at 37°C (Fig 9A). The *efg1* mutant was hyperfilamentous at 25°C but non-filamentous at 37°C verifying the previously reported dual repressor/activator role of Efg1 [19,23,29]. Strong hypha formation was also observed for the *bcr1* mutant at 25°C but unlike the *efg1* mutant, this mutant had a hyperfilamentous phenotype at 37°C. Both *ace2* and *brg1* mutant were defective in filamentation; this defect occurred for the *ace2* mutant in all conditions, whereas the *brg1* mutant was able to filament at 37°C in the presence of CO_2_. Interestingly, under normoxia the wild-type strain presented vigorous filamentation at 37°C, while all single mutants showed complete or partial (*ace2* mutant) filamentation defects (S8 Fig). Thus, the Efg1, Ace2, Bcr1 and Brg1 regulators determine morphogenesis under both hypoxia and normoxia.

To establish if the hyperfilamentous phenotype of the *efg1* and *bcr1* mutants at 25°C requires Ace2 and/or Brg1 proteins double mutants were constructed and tested (Fig 9B). The construction of a homozygous *efg1 ace2* double mutant failed repeatedly suggesting that a *C*. *albicans* strain lacking both Efg1 and Ace2 is not viable; therefore, an *ace2/ace2 efg1/EFG1* heterozygous mutant (CLvW047) was constructed. Filamentation of the *efg1/EFG1* heterozygote was slightly but reproducibly increased at 25°C as compared to the control strain, while filamentation was reduced in strain CLvW047. The hyperfilamentous phenotype of the *bcr1* mutant (especially in the presence of CO_2_) was also reduced in the *bcr1 ace2* double mutant. These results suggest that the increased hypha formation of *efg1* and *bcr1* mutants requires Ace2. The Brg1 protein is also needed for this phenotype because *efg1 brg1* and *bcr1 brg1* double mutants were completely defective for filamentation at 25°C; at 37°C, Brg1 was also needed for the *bcr1* phenotype in the absence of CO_2_. In summary, the results indicate that hyphal morphogenesis of *C*. *albicans* under hypoxia is effectively repressed by Efg1 and Bcr1, counteracting the stimulatory effects of the Ace2 and Brg1 proteins.

## Discussion

*C*. *albicans* is an opportunistic pathogen that inhabits the human host as a harmless commensal but that also can turn into a serious pathogen, which causes tenacious superficial and deadly systemic fungal disease. Candidiasis is typically caused by the strong proliferation of the same *C*. *albicans* strain that had inhabited the patient as a commensal before [6] raising questions about the molecular events that occur in the pathogen during the commensal-to-pathogen transition. As a commensal, *C*. *albicans* colonizes the gut and partly also mucosal surfaces [2,71–73]. Recent results have suggested that the fungus actively restrains its proliferation in the gut by transcriptional regulators Efg1 and Efh1 [7,8,74], while the Wor1 protein enhances gut colonization [9]. Events in the gut occur in oxygen-poor conditions (partly under anoxia) and mostly at elevated carbon dioxide concentrations [14,15]. Here we describe a transcriptional hub that downregulates filamentous growth of *C*. *albicans* and favors proliferation of its yeast form under hypoxic conditions. Surprisingly, this repressive activity involves regulators including Efg1, which positively regulate filamentation under normoxia.

Efg1 directs several aspects of morphogenesis and metabolism in *C*. *albicans*. It has an important transcriptional role under hypoxia since it contributes to but also prevents hypoxic regulation of many genes [14,19]. The change to a hypoxia-specific pattern of gene expression requires Efg1 at an early time-point following a shift to hypoxia [25]. It is known that Efg1 has a dual role on hyphal morphogenesis: under hypoxia it acts as a hyphal repressor during growth on agar at temperatures ≤ 35°C [19,23,28], while under normoxia, Efg1 is a strong inducer of hypha formation [26,27]. The Efg1 signaling pathway under normoxia comprises adenylate cyclase Cyr1 activity that increases cAMP levels [46,47], which activates PKA isoforms Tpk1/Tpk2 and in turn Efg1 by phosphorylation of residue T206 [39]; an additional phosphorylation of T179 by the Cdc28-Hgc1 complex was also described to occur during hyphal morphogenesis [33]. Here we report that the hypoxic repressor function of Efg1 has specific structural requirements. Efg1 lost its repressor activity, when its N-terminal end was modified by extension and partially by deletion, while under normoxia such variants were active in hyphal induction [38]. Interestingly, chlamydospore formation, which is induced by oxygen limitation, also was found to require an undeleted N-terminus of Efg1 [28]. In addition, phosphomimetic residues at Efg1 phosphorylation sites (T179E, T206E) blocked the hypoxic repressor activity, while the corresponding alanine replacement variants were fully active in repression but inactive for the normoxic induction of hyphae [33,39]. This result corresponds to the lowered *CYR1* and *TPK1* transcript levels under hypoxia, which predicts lowered PKA activity and reduced T206 phosphorylation, thus resulting in enhanced hypoxic repressor activity of Efg1. With regard to Efg1 target sequences, the deduced CA-rich hypoxic binding sites did not match the major normoxic binding site TATGCATA for the yeast growth form, although Efg1 binds to CA- sequences shortly after hyphal induction [40]. Thus, the different functions of Efg1 as a hypoxic repressor involve different recognition and target sequences, as compared to normoxia.

**Fig 10:**
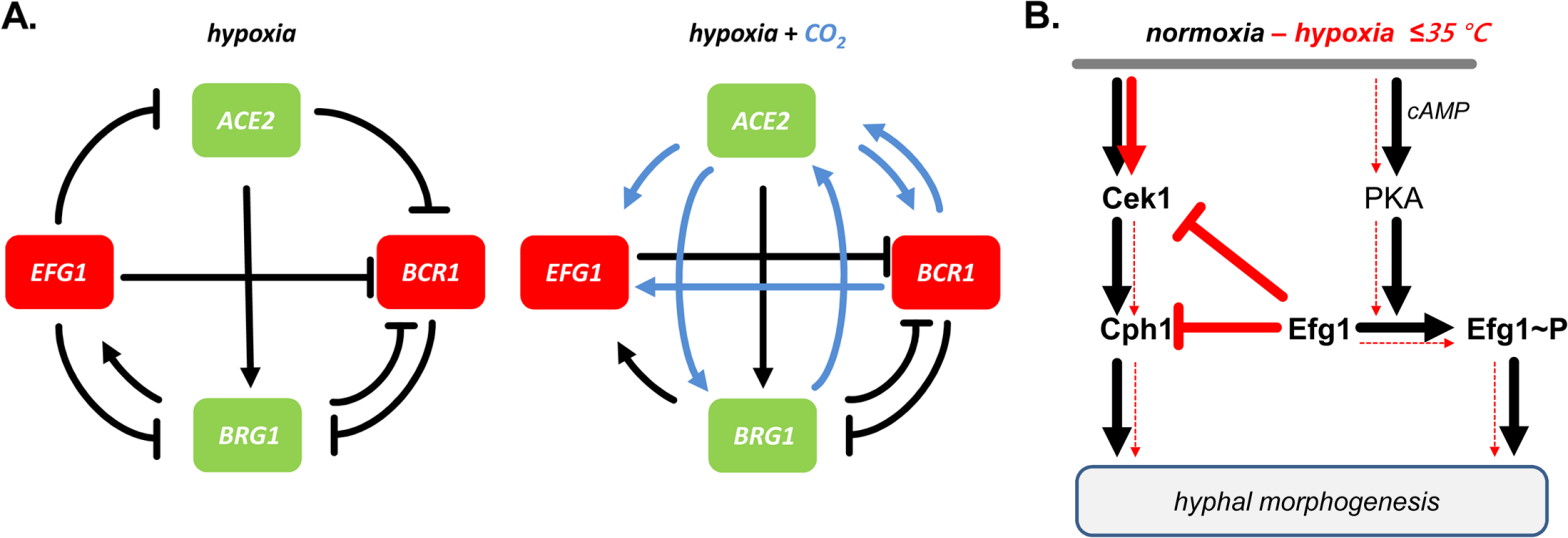
Models for transcriptional regulation and morphogenesis under hypoxia. (A) Transcriptional circuits. Hypoxic repressors Efg1 and Bcr1 (red) and hypoxic activators Ace2 and Brg1 (green) are mutually connected in regulatory circuits to regulate hypha formation of *C*. *albicans*. Note that regulatory circuits differ depending on the availability of CO_2_ (blue arrows). (B) Morphogenetic signaling pathways. Under normoxia both Cek1 and PKA kinase pathways trigger hypha formation; Efg1 phosphorylation provides a major signalling input. Under hypoxia, at temperatures ≤ 35°C, low levels of Efg1 phosphorylation block the Cek1 pathway, thereby preventing hyphal induction by both pathways.

Previously, synergistic and antagonistic functions of Efg1 and Ace2 transcription factors have been described. Both proteins enhance glycolytic and oxidative patterns of gene expression [23,30] and positively influence filamentation under normoxia [26,27,30–32]. In contrast, under hypoxia Efg1 represses hypha formation [19,23,28], while Ace2 acts as an inducer [30,32]. Efg1 represses *ACE2* transcript levels, possibly by direct binding of Efg1 to the *ACE2* promoter [32]. To further characterize the functional intersection of both regulators we used ChIP chip analyses to compare their genomic binding patterns under hypoxia. A significant overlap of target genes was identified and the deduced Ace2 binding sequences in promoters included sequences resembling the ACCAGC motif for *S*. *cerevisiae* Ace2 [50] but also the above discussed CA-sequences representing hypoxic binding sites for Efg1. Interestingly, the group of genes targeted by both (HA-)Efg1 and Ace2 included important regulators of hyphal growth, biofilm formation and cell adhesion. Targets included the *EFG1* promoter, which thereby was confirmed not only as an autoregulatory target for Efg1 [40] but also identified as an Ace2 target. Confirming this result, Ace2 was required for upregulation of the *EFG1* transcript in a hypoxic CO_2_-containing atmosphere, while Efg1 repressed the *ACE2* transcript as under normoxia [32]. We analyzed the mode of joint target gene regulation by the Efg1/Ace2 proteins by focusing on *BCR1* [52,66,67] and *BRG1 (GAT2)* [51,68,69], which regulate filamentation and were found to get hypoxically down- and, respectively, upregulated. Surprisingly, these genes were not only targets but also regulators of Efg1/Ace2 and they negatively regulated each other, thereby generating an interconnected regulatory loop (Fig 10A). Efg1 acted as hypoxic repressor of *BCR1/BRG1* and also of two other target genes (*TYE7*, *ZCF21*), while it was an inducer of *AAF1* expression (S3 Fig). In general, Ace2 activated hypoxic expression of all of these genes, especially in the presence of CO_2_. Transcripts of hypoxia-upregulated genes including *EFG1*, *BRG1* and *TYE7* and of hypoxia-downregulated genes including *BCR1*, *AAF1* and *ZCF21* were all reduced if *ace2* mutant cells were grown hypoxically in the presence of CO_2_. Interestingly, Ace2 bound strongly to the promoter of the *FLO8* gene, which encodes a CO_2_ sensor interacting with Efg1 [38,62] that is required for white-to-opaque switching and for filamentous growth [61]. Thus, the lack of oxygen combined with an increased level of CO_2_ generates an environment that elicits a specific regulatory response in *C*. *albicans*. These results are reminiscent of and confirm previous results for the hypoxia-specific, CO_2_-dependent functions of Sch9 kinase [37] and the Ume6 regulator [75].

The morphogenetic output of the described hypoxic regulatory hub was tested by examining hypha formation on agar. The results confirmed that Ace2 is a positive factor for filamentation under hypoxia and partly also under normoxia [30,32], while Efg1 has a dual repressor/activator function under hypoxia/normoxia. Similar to Efg1, Bcr1 acted as a repressor of hypha formation at 25°C, especially in the presence of CO_2_. Transcript data suggested that in this environment, Ace2 stimulates *BCR1* expression to ensure efficient blockage of hypha formation. Brg1 was needed for hypha formation at 37°C under hypoxia, but only in the absence of CO_2_; in its presence, Brg1 was dispensable for filamentation. The *BRG1* transcript was repressed by Efg1 and activated by Ace2, largely independent of CO_2_. As shown by the phenotypes of double mutants the increased filamentation of *efg1* and *bcr1* mutants depended on the activity of both Brg1 and Ace2. Thus, under hypoxia *C*. *albicans* restrains the stimulatory actions of both Brg1 and Ace2 on hyphal morphogenesis, using Efg1 and Bcr1 as repressors. While this study puts its focus on hypha formation, it is likely that other adaptation processes occurring under hypoxia, especially re-direction of metabolism to a fermentative mode, are regulated by transcription factors that link to the Efg1-Ace2 regulatory hub. Relevant transcription factors for this function could include Tye7 [56], Aaf1 [53] and Zcf21 [60], proteins that regulate glyolysis, biofilm formation and/or commensalism.

Which signaling pathway of morphogenesis is downregulated by Efg1 or Bcr1 proteins? Transcript analyses revealed that the genes encoding MAP kinase Cek1, its downstream target Cph1 and the kinase Kic1 are downregulated specifically under hypoxia by Efg1. The Cek1/Cph1 pathway is known to permit filamentation under normoxia during surface growth of *C*. *albicans* [43–45], while in *S*. *cerevisiae*, Kic1 is part of the RAM pathway that activates Ace2 activity [48]. The repressive action of Efg1 and its co-regulators on the Cek1 pathway was verified by demonstrating that Efg1, Ace2, Bcr1 and Brg1 all act as repressors of *CEK1* transcript levels, while the *CPH1* transcript was especially repressed by Bcr1 and Efg1. Furthermore, Efg1- and Ace2-mediated repression of the Cek1 protein in its non-phosphorylated and phosphorylated form was also demonstrated. Confirming these results, an *efg1 cph1* double mutant was unable to filament, while overexpression of *CPH1* in a wild-type genetic background triggered hypha formation under hypoxia. These results clearly indicate that *C*. *albicans* actively suppresses filamentation mediated by the Cek1 pathway in certain oxygen-poor conditions to promote proliferation of the yeast form (Fig 10B). We have discovered this suppression *in vitro* slightly below the core body temperature (i. e. < 37°C) but this activity may also occur in the special molecular environment of the gastrointestinal tract or in hypoxic skin tissue [16,17]. Nevertheless, this scenario does not exclude that upregulation of hypoxic filamentation may occur under hypoxia, e. g. if the repressive action of Efg1 is blocked by the Czf1 protein [4]. In this situation, other regulators including Mss11 and Rac1, which were identified by their embedded growth phenotypes [76,77], may also promote hypoxic filamentation. A similar hyperfilamentous phenotype was also reported for mutants lacking the kinase Sch9, although elevated CO_2_-levels were required in this case [37]. In the human gut, locally increased filamentation could favor anchoring of *C*. *albicans* to the epithelium and trigger strong fungal proliferation and systemic invasion mediated by invasion-specific regulators including Eed1 [8,55]. These events may initiate the pathogenic stage of fungal colonization, which will ultimately become apparent by the symptoms of disease. It appears that as a commensal *C*. *albicans* attempts to avoid immune responses and to maintain its residency by downregulation of filamentation and possibly other virulence traits. Strengthening of fungal commensalism, e. g. by novel therapeutic molecules or by probiotic microbes could become a promising strategy to combat serious fungal disease.

## Materials and Methods

### Strains and media

*C*. *albicans* strains are listed in S4 Table. Strains were grown in liquid YP medium (1% yeast extract, 2% peptone) containing 2% glucose (YPD) or 2% sucrose (YPS); solid media contained 2% agar. An Invivo200 hypoxia chamber (Ruskinn) was used for hypoxic growth under 0.2% O_2_ [37]; liquid media were pre-equilibrated overnight under hypoxia before inoculation. Strains overexpressing genes using tetracyclin-inducible promoters were grown in/on YPS medium containing 3 μg/ml anhydrotetracycline.

### *C*. *albicans* strains expressing *EFG1* variants

Oligonucleotides are listed in S5 Table. Plasmid pTD38-HA contains promoter and coding region for an N-terminally hemagglutinin (HA)-tagged Efg1 [38]. In this plasmid, HA-encoding sequences reside on a *Bgl*II and *Bam*HI fragment, which were removed in plasmid pPRDEFG1, in which the native *EFG1* ORF is preceded by a *Bam*HI site. Other plasmids carrying *EFG1* genes encoding Efg1 variants without HA tag were constructed in two steps, as in the case of pPRDNEFG1, in which nucleotides 25 and 222 of the *EFG1* ORF are deleted encoding a variant lacking residues Y9 to G74 of Efg1. Primers EFG1BamHIFor and EFG1BamHIRev were used for PCR amplification of the ORF of this variant, using plasmid pBI-HAHYD-D1 [38] as the template. The *Bam*HI-digested PCR fragment was inserted downstream of the *EFG1* promoter by ligation with the large *Bgl*II fragment of pTD38-HA to generate plasmid p2621NΔEFG1. The *Pac*I-*Spe*I fragment of this plasmid carrying the junction of the *EFG1* promoter and its ORF was then used to replace the corresponding fragment in pTD38-HA. By this procedure, the mutated *EFG1* ORF was joined to its 3´-UTR. Similarly, the mutated *EFG1* alleles encoding the T206A and T206E mutations were transferred from plasmids pDB1 and pDB2 [39] into pTD38-HA to generate plasmids pPDEFG1T206A and pPDEFG1T206E. Analogous plasmids encoding T179A and T179E Efg1 variants were constructed by mutating the *EFG1* ORF by primer pairs EFG1179AFor/Rev and T179Efor/rev, respectively, using site-directed mutagenesis (QuikChange kit, Stratagene). The resultant plasmids pPDEFG1T179E and pPDEFG1T179A encode T179A and T79E variants of Efg1, respectively. All constructs were sequenced using primers EFG1seqFor, EFG1seqRev and Efg1SeqM to confirm the presence of the mutations within *EFG1*.

Plasmids were integrated into the chromosomal *EFG1* locus of *efg1* mutant HLC67 by transformation following digestion with *Pac*I in the *EFG1* promoter [38]. Their correct chromosomal integration was verified by PCR of gDNA using primers UTREfg1For and Efg1seqM.

### *C*. *albicans* strains expressing HA-tagged Ace2

For localization studies of Ace2 a heterozygous mutant strain was constructed first. One *ACE2* ORF was replaced with a *lac*Z-*ACT1p*-*SAT1* cassette. The cassette was amplified from plasmid pStLacZ-SAT. All PCR products were separated on an agarose gel and purified using the QIAquick Gel Extraction Kit (Qiagen) before transformation. The oligonucleotides (lacZACE2for/lacZACE2rev) used for amplification, tagged the cassette with 90 bp of flanking sequence complementary to the *ACE2* ORF. The strain BWP17 was transformed and transformants were screened for nourseothricin resistance. Integration of the *lac*Z-*ACT1p*-*SAT1* cassette and replacement of the *ACE2* ORF was verified by Southern blot analysis using a probe for *SAT1*. The resulting heterozygous strain CLvW001 (*ace2*-*lac*Z-*ACT1p*-*SAT1*/*ACE2*) was used for C terminal tagging of Ace2. Oligonucleotides ACE2HAFor and ACE2HARev were used to amplify the triple HA-encoding sequence from plasmid p3HA-URA3 thereby adding homologous sequences of the 3‘- end of the *ACE2* ORF and 5‘-UTR of the *ACE2* allele to the amplicon. Strain CLvW001 was transformed for C terminal tagging of the remaining *ACE2* allele. Correct chromosomal integration at the *ACE2* locus was confirmed by colony-PCR using oligonucleotides ACE2For and HARev and by Southern blot analysis. The resulting strain CLvW004, expressing C-terminal HA-tagged Ace2 at the native locus was used to study chromosomal localization of Ace2-HA.

### Construction of *ACE2* deletion strains

For the deletion of *ACE2* in the *bcr1* and *efg1* mutant background and in strain CAI4, plasmid pSFS5 was used, containing a modified *SAT1* flipper cassette [78]. The *ACE2* upstream and downstream regions were amplified with the oligonucleotide pairs CAF1ApaI/CAF2XhoI and CAR1SacII/CAR2SacI, for construction of the inner deletion cassette, and oligonucleotide pairs CAF3ApaI/CAF4XhoI and CAR3SacII/CAR4SacI for construction of the outer deletion cassette; the *ace2* mutant strain MK106 described by Kelly *et al*. [24] was used as the reference strain. gDNA of strain SC5314 was used to generate PCR products, which were digested with the indicated restriction enzymes and cloned on both sites of the *SAT1* flipper cassette in pSFS5 [78] resulting in plasmids pCLvW90 (inner cassette) and pCLvW91 (outer cassette). The deletion cassettes in plasmids pCLvW90 and pCLvW91 were released by digestion with *Sac*I and *Apa*I and used for transformation of strains using first the fragment containing the outer cassette and, after excision of the *SAT1* flipper cassette, with the second fragment containing the inner cassette. Integration of the deletion cassette was confirmed by colony PCR using oligonucleotides FLP1, which binds within the *FLP1* gene, and ACE2UTR5, which binds upstream of the *ACE2* ORF. Transformants containing the *ACE2* deletion cassette were grown overnight in liquid YCB-BSA medium (20 g yeast carbon base, 4 g bovine serum albumin and 2 g yeast extract per liter) to induce excision of the cassette by FLP-mediated recombination; corresponding strains were identified by their small colony size on YPD plates containing 25 μg/ml nourseothricin. After excision of the second *SAT1* flipper cassette, deletion of both *ACE2* alleles was confirmed by Southern blot analysis. With this approach both *ACE2* alleles were deleted in the *bcr1* mutant strain CJN702 [52] resulting in strain CLvW024 and in CAI4 resulting in strain CLvW008. However, deletion of the second *ACE2* allele in the *efg1* mutant HLC52 [27] was not successful.

In an additional approach we tried to generate the *ace2 efg1* double knockout strain by deleting *EFG1* in the *ace2* mutant strain CLvW008 with the Ura-blaster disruption technique [79] but again we were not able to obtain a homozygous mutant strain suggesting that the double knockout is lethal. The *URA3* deletion cassette used for this approach was released from plasmid pBB503 [80] by digestion with *Hin*dIII and *Kpn*I. The fragment was purified and transformed into strain CLvW008 and transformants were selected for uridine prototrophy on SD agar. Integration of the cassette was confirmed by colony-PCR. In the resulting strain CLvW041 one chromosomal copy of *EFG1* was replaced by the sequence of *URA3* flanked by *hisG* sequences (*efg1*::*hisG*-*URA3*-*hisG*/*EFG; ace2*::*FRT*/*ace2*::*FRT*). Attempts to delete the second *EFG1* allele after removal of *URA3* [79] were not successful.

### Construction of *BRG1* deletion strains

For deletion of *BRG1* gene in *efg1* (HLC52) and *bcr1* (CJN702) mutants the upstream region of the *BRG1* ORF was amplified by genomic PCR using primers BRG1 5UTRKpnIFor/KpnIRev and cloned into the *Kpn*I site of plasmid pSF5S to generate pSFS5-B5. The *BRG1* downstream region was amplified using primers BRG13UTRNot1For/SacIRev and cloned into *Not*I and *Sac*I sites of pSFS5-B5 to generate pSFS5-B5B3. The *Kpn*I-*SacI* fragment of this plasmid was used to transform *efg1* and *bcr1* mutant strains, selecting transformants on YPD agar containing nourseothricin (200 μg/ml). Correct genomic integration was confirmed by colony PCR using oligonucleotides FLP1, which binds within the *FLP1* gene, and BRG1UpFor, which binds upstream of the *BRG1* promoter region. Verified heterozygous transformants were grown in YCB-BSA medium to evict the disruption cassette and retransformed with the disruption fragment, as described [78]. Deletion of both *BRG1* alleles was confirmed by negative colony PCR using primer BRG1UpFor, which binds upstream to the *BRG1* ORF and BRG1midrev that is specific for the *BRG1* ORF. *BRG1* alleles were deleted in *efg1* (HLC52) and *bcr1* (CJN702) mutant strains resulting in strains PDEB4 (*efg1 brg1*) and PDBB4 (*bcr1 brg1*) respectively.

### *C*. *albicans* strains overproducing signalling components

The plasmids pClp10TETSTE11, pClp10TETCEK1 and pClpTETCPH1 [81] encoding Ste11, Cek1 and Cph1 proteins were linearized with *Stu*I within the *RPS1* sequence and transformed into *C*. *albicans* CEC2907 [81] selecting for uridine prototrophy; the resultant strains were named CECSTE11, CECCEK1 and CECCPH1. Correct plasmid integration at the *RPS1* locus was confirmed by colony PCR using primers ClpUL and ClpUR.

### Generation of an anti-Efg1 antiserum

A rabbit polyclonal anti-Efg1 antiserum was generated using His_10_-tagged Efg1 produced in *E*. *coli*. The *EFG1* ORF (allele *ORF19*.*8243*) residing on a *Xho*I-*Bam*HI fragment was subcloned into pET19b (Novagen), downstream of the T7 RNA polymerase promoter. The resulting plasmid encoded a His_10_-Efg1 fusion but contained a single CUG codon (residue 449) that encodes serine in *C*. *albicans* but leucine in *E*. *coli*. This codon was changed to a UCG serine codon by site-directed mutagenesis using oligonucleotides pET19Serinhin/her, resulting in plasmid pET19-His-Efg1Kodon, which was transformed into *E*. *coli* Rosetta 2 (DE3)pLysS (Merck). Transformants were grown and the T7 promoter was induced according to instructions of the manufacturer. Cells were resuspended in buffer (20 mM CAPSO pH 9.5, 1 M NaCl, 1 mM EDTA, 20 mM imidazol, 0.1% Triton X100) and broken using 3 passages through a French press cell (Slaminco Spectronic Instruments). Crude extracts were cleared by centrifugation and applied to HisTrap columns connected to an ÄKTA prime plus fraction collector (GE Healthcare). The His_10_-Efg1 fusion protein was eluted using CAPSO buffer containing 250 mM imidazol. Purified protein (100 μg) was injected on days 1, 14, 28 and 56 in 2 New Zealand White rabbits (performed by Eurogentec, Belgium). One rabbit generated high anti-Efg1 titers in ELISA tests and in immunoblottings (dilution 1:5000).

### Immunodetection

YPD precultures were grown under normoxia overnight at 30°C in YPD medium and were used to inoculate 40 ml of YPD medium, which had been preincubated overnight under hypoxia (0.2% O_2_). Starting with an initial density of OD_600_ = 0.1 cells were grown at 30°C under hypoxia to an OD_600_ = 1. Cells were harvested, frozen at -70°C for 1 h and then thawed by addition of 500 ml of CAPSO buffer (20 mM CAPSO pH 9,5, 1 M NaCl, 1 mM EDTA, 20 mM imidazole, 0,1% Triton X-100) containing protease inhibitor (Cocktail Complete, Mini, EDTA-free/Roche). Cells were broken at 4°C by shaking with one volume of glass beads (0.45 mm) in a FastPrep-24 shaker (MP Biomedicals) using 4–6 cycles for 40 s at 6.5 ms^-1^; between cycles cells were placed on ice for 5 min. Debris was removed by centrifugation at 13,000 rpm for 5 min and protein in the supernatant was determined using the Bradford assay. 45 μg of the crude cell extract was separated by SDS-PAGE (8% polyacrylamide) and analysed by immunoblotting using anti-Efg1 antiserum (1:5,000) or anti-histone H4 (Abcam; 1:5,000) to detect histone H4 as loading control. Total Cek1 levels were detected by immunoblotting using anti-Cek1 antiserum [10], while phosphorylated Cek1 was detected using monoclonal rabbit anti-phospho-p44/42 antibody (Cell Signaling Technology). Anti-rabbit-IgG-HRP conjugate (1:10,000) was used as secondary antibody in all blottings. Signals generated by the chemiluminescent substrate (SuperSignal West Dura; Pierce) were detected by a LAS-4000 mini imager (Fujifilm) and evaluated by the Multi Gauge Software (Fujifilm).

### Chromatin immunoprecipitation on microchips (ChIP chip)

The ChIP chip procedure was performed as described by Lassak *et al*. [40], except that the strains and antibodies used for immunoprecipitation were different. Two independent cultures were assayed for each combination of strains. Precultures were grown overnight under normoxia at 30°C in YPD medium and were shifted to YPD medium precalibrated under hypoxia (0.2% O_2_, 30°C). The cells were allowed to grow from OD_600_ = 0.1 to 1. Two sets of strains were analysed: (1) wild-type strain CAF2-1 as test strain and *efg1* mutant HLC52 as control strain were compared to determine the genomic localization of untagged Efg1, using anti-Efg1 antibody for chromatin immunoprecipitation; (2) Strain HLCEEFG1 producing HA-tagged Efg1 as test strain and DSC11 (Efg1 producing) as control strain were compared to determine the genomic localization of HA-Efg1 using anti-HA antibody for chromatin immunoprecipitation. *C*. *albicans* genomic tiling microarrays were probed pairwise by immunoprecipitated chromatin as described previously [40]. For localization studies of Ace2, precultures were grown overnight under normoxia at 30°C in YPD medium and were used to inoculate medium preincubated under normoxia or hypoxia (0.2% O_2_) with addition of 6% CO_2_. Strain CLvW004, producing HA-tagged Ace2 from its native locus and control strain BWP17 were used to determine the genomic localization of Ace2-HA using anti-HA antibody for immunoprecipitation. Significant binding peaks were defined as probes containing four or more signals above background in a 500 bp sliding window; the degree of significance depended on the FDR value. Results were visualized using the program SignalMap (version 1.9). The most significant binding peaks (FDR ≤ 0.05) for Efg1 (202 peak genomic binding sites), HA-Efg1 (106 peak genomic binding sites) and Ace2-HA (272 peak genomic binding sites), which coincided in both replicates, were analysed by the program RSAT dyad-analysis to predict DNA binding sequence [40].

## Supporting Information

S1 Fig Activity of rabbit anti-Efg1 antiserum. (A) Immunoblotting. Extracts of *efg1* mutant HLC67 (lane 2) and control strain CAF2-1 (lane 3) were separated by SDS-PAGE and blots were probed with anti-Efg1 antiserum (1: 5000). For comparison, 250 ng of *E*. *coli-*produced His_10_-Efg1 protein was used (lane 1). (B) Immunoprecipitation. Efg1 was immunoprecipitated using anti-Efg1 antiserum and protein G-coated agarose beads. Immunoprecipitates from the control strain CAF2-1 (lane 1) and the *efg1* mutant HLC67 (lane 2) were analysed by immunoblotting using anti-Efg1 antiserum. The arrow indicates the migration of Efg1.
(PDF)

S2 Fig Intersection of genomic binding sites for Efg1 and HA-Efg1 under hypoxia and normoxia. For Efg1, genomic binding sites were derived from ChIP chip experiments comparing strains CAF2-1 (*EFG1/EFG1*) and HLC52 (*efg1*/*efg1*); for HA-Efg1, strains HLCEEFG1 (*efg1*/*efg1* [*HA-EFG1*]) and CAF2-1 were compared. Normoxic binding sites for HA-Efg1 were obtained from Lassak *et al*. [40]. The shaded circle encompasses genes in filamentous growth.
(PDF)

S3 Fig GO categories of genes binding HA-Efg1 under hypoxia. GO terms for Efg1 binding targets were identified in ChIP chip data using the CGD GO Term Finder tool (http://www.candidagenome.org/cgi-bin/GO/goTermFinder); the analysis was conducted in June 2013. Genome frequencies of genes corresponding to GO terms are expressed as percentages (gene number relative to 6,525 genes in the *C*. *albicans* genome; the frequency of genes binding Efg1 that correspond to a specific GO term are expressed relative to the total number of 106 genes binding HA-Efg1). Superscripts: a, Efg1 binding in yeast normoxia [40]; b, Efg1 binding in hyphae inducing conditions [40]; c, Efg1 binding in biofilm inducing conditions [42]. *P* values for overrepresented categories were calculated using a hyper geometric distribution with multiple hypothesis correction according to the GO Term Finder tool website (http://www.candidagenome.org/help/goTermFinder.shtml). The *P* value cutoff used was 0.05.
(PDF)

S4 Fig Functionality of the HA tagged Ace2 protein. To verify the functionality of C-terminal HA-tagged Ace2 the phenotypes of three isolates of strain CLvW004 (*ACE2*-*HA*/*ace2*) was compared to mutant strain MK106 (*ace2*/*ace2*), wild-type strain BWP17 (*ACE2*/*ACE2*) and the heterozygous strain CLvW001 (*ACE2*/*ace2*). (A) Drop dilution assay for sensitivity to 4 μM Pmt1-inhibitor [49] and resistance to antimycin A (20 μg/ml). The agar plates were photographed after 2 d incubation at 30°C. The *ace2* mutant strain Mk106 shows enhanced sensitivity to the Pmt1-inhibitor and is less susceptible to the respiratory inhibitor antimycin A [30]. (B) Colonies of the indicated strains were photographed following growth for 2 d at 30°C on YPD agar. The *ace2* mutant strain Mk106 shows the wrinkled colony phenotype described previously [24]. Phenotypes of strains CLvW004.1–3 correspond to the heterozygous strain CLvW001 indicating that the Ace2-HA protein in these strains is functional.
(PDF)

S5 Fig Genomic binding sites for Ace2 under hypoxia and normoxia. Venn diagram showing numbers of genes bound by HA-tagged Ace2 under normoxic conditions (30°C, YPD) and hypoxic conditions (30°C, YPD 0.2% O_2_ and 6% CO_2_). Binding regions with the corresponding ORFs are listed in S2 and S3 Table. Genomic binding sites were derived from ChIP chip experiments comparing strains ClvW004 (*ACE2-HA/ace2*) and BWP17 (non tag control).
(PDF)

S6 Fig Genomic localization of Ace2 and Efg1 on target promoters. (A) Binding positions identified by ChIP chip experiments. Binding positions of Efg1 (black ovals), Ace2-HA (orange ovals) and HA-Efg1 positions (grey ovals) identified by ChIP chip experiments are shown schematically. (B) Quantitation of Ace2, HA-Efg1 and Efg1 enrichment by ChIP on target promoters. Following ChIP the respective fold enrichment was determined by qPCR using oligonucleotide pairs shown by red arrows in A. Chromatin of strains CAF2–1 (Efg1) and HLCEEFG1 (HA-Efg1) was immunoprecipitated using anti-Efg1 and anti-HA antibody, respectively, as described in S1 Table. For Ace2 enrichment anti-HA antibody was used for immunoprecipitation of strain CLvW004 (Ace2-HA), as described in S2 Table. In a control experiment, ChIP followed by qPCR was also done using anti-Efg1 antibody on extracts of strain HLCEEFG1 (HA-Efg1). qPCR experiments were done using two biological replicates, which were assayed in triplicate. The mean fold enrichment (± standard deviation) for each protein was calculated relative to the respective no tag or mutant strain and normalized to the input sample. Statistical relevance is indicated by asterisks: *, *P* < 0.05; **, *P* < 0.01; ***, *P* < 0.001. The results verify the presence of proteins on target promoters; in addition, the presence of HA-Efg1 on the *BCR1* promoter, which was not found in the ChIP chip experiment, was revealed by the ChIP-qPCR experiment.
(PDF)

S7 Fig Transcriptional regulation of selected Efg1 and Ace2 target genes under hypoxia. Strains CAF2-1 (control), the *ace2* mutant MK106 and the *efg1* mutant HLC52 were precultured under normoxia at 30°C in YPD medium and used for inoculation of 100 ml YPD cultures. Cultures were incubated at 30°C under normoxia, or normoxia with addition of CO_2_ (6% CO_2_), or under hypoxia (0.2% O_2_), or under hypoxia with addition of CO_2_ (0.2% O_2_, 6% CO_2_) until OD_600_ = 0.5 and total RNA was then isolated. Relative transcript levels were determined using *ACT1* transcript as the reference as described in Fig 4. Relative transcript levels (RTL) for the *AAF1*, *TYE7* and *ZCF21* transcripts in strains CAF2-1 (control), HLC52 (*efg1*) and MK106 (*ace2*). Error bars represent standard deviation of the means. A two-tailed, unpaired *t* test comparing the cycle threshold values of samples grown in hypoxic and normoxic conditions for each mutant respectively was used to determine the statistical relevance: *, *P* < 0.05; **, *P* < 0.01; ***, *P* < 0.001.
(PDF)

S8 Fig Normoxic phenotypes of mutants lacking hypoxic regulators under hypoxia. The strains were grown under normoxia without or with 6% CO_2_ on YPS agar for 4 d at 25°C or at 37°C for 3 d. Strains included CAF2-1 (control), homozygous single mutants HLC52 (*efg1*), CJN702 (*bcr1*), MK106 (*ace2*), TF022 (*brg1*) and double knockout strains PDEB4 (*efg1 brg1*), PDBB4 (*bcr1 brg1*) and CLvW024 (*bcr1 ace2)*. *ACE2* could not be disrupted in an *efg1* mutant background; therefore, the heterozygous mutant strain CLvW047 (*efg1/EFG1*, *ace2*/*ace2*) was constructed and its phenotype was compared to the *efg1/EFG1* strain DSC11.
(PDF)

S1 Table Binding of Efg1 to chromosomal sequences of *C*. *albicans* under hypoxia. Strain CAF2-1 producing wild-type Efg1 was grown in YPD medium at 30°C under hypoxic conditions (0.2% O_2_), chromosomal cross-linking of proteins was done. Fragmented chromatin was immunoprecipitated using anti-Efg1 antibody and used as probe for tiling microarrays covering the *C*. *albicans* genome. Regions showing significantly increased binding of Efg1 compared to control strain HLC52 (*efg1/efg1*) are listed along with their nearest neigbouring ORFs designated left or right ORF if situated at descending and, respectively, ascending chromosomal coordinates. ORF orientations are indicated by the arrows. Other proteins binding to the same chromosomal region are also indicated. Binding regions within coding regions are marked in purple lettering and green shading indicates multiple binding regions. *, significant binding regions that do not overlap in both replicates.
(XLSX)

S2 Table Binding of Ace2-HA to chromosomal sequences of *C*. *albicans* under hypoxia and CO_2_. Strain CLvW004 producing Ace2-HA from its chromosomal ORF and reference strain BWP17 were grown in YPD medium at 30°C under hypoxic conditions and in presence of elevated CO_2_ levels (0.2% O_2_ and 6% CO_2_). Chromosomal cross-linking of proteins was done and chromatin was fragmented before immunoprecipitation. For immunoprecipitation anti-HA antibody was used and the precipitated chromatin was spotted on a *C*. *albicans* whole-genome tiling microarray. Regions showing significant enrichment of Ace2-HA binding compared to control strain BWP17 are listet along with their nearest neighbouring ORFs. Other proteins binding to the same chromosomal region are indicated and are taken from Lassak *et al*. [40]. Binding targets of wild-type Efg1 or HA-Efg1 shared with Ace2-HA are indicated with red background color. Accordingly to transcriptomal data obtained by Mulhern *et al*. [30], up- and down-regulation of genes is indicated of identified Ace2-HA target genes.
(XLSX)

S3 Table Binding of Ace2-HA to chromosomal sequences of *C*. *albicans* under normoxia. Strain CLvW004 producing Ace2-HA from its chromosomal ORF and reference strain BWP17 were grown in YPD medium at 30°C under normoxic conditions. Chromosomal cross-linking of proteins was done and chromatin was fragmented before immunoprecipitation. For immunoprecipitation anti-HA antibody was used and the precipitated chromatin was spotted on a *C*. *albicans* whole-genome tiling microarray. Regions showing significant enrichment of Ace2-HA binding compared to control strain BWP17 are listet along with their nearest neighbouring ORFs. Other proteins binding to the same chromosomal region are indicated and are taken from Lassak *et al*. [40]. Binding targets of wild-type Efg1 or HA-Efg1 shared with Ace2-HA are indicated with red background color, binding targets of Ace2-HA obtained uniquely under normoxic conditions are indicated with green background color. Accordingly to transcriptomal data obtained by Mulhern *et al*. [30], up- and down-regulation of genes is indicated of identified Ace2-HA target genes.
(XLSX)

S4 Table *C*. *albicans* strains. (PDF)

S5 Table Oligonucleotides. (PDF)
