# Supplementary material for: Hypoxia and Temperature Regulated Morphogenesis in Candida albicans
Source: PLoS Genet. 2015 Aug 14;11(8):e1005447. doi: 10.1371/journal.pgen.1005447 (PMC4537295; doi:10.1371/journal.pgen.1005447)
Supplement: S1 Fig — (A) Immunoblotting. Extracts of efg1 mutant HLC67 (lane 2) and control strain CAF2-1 (lane 3) were separated by SDS-PAGE and blots were probed with anti-Efg1 antiserum (1: 5000). For comparison, 250 ng of E. coli-produced His10-Efg1 protein was used (lane 1). (B) Immunoprecipitation. Efg1 was immunoprecipitated using anti-Efg1 antiserum and protein G-coated agarose beads. Immunoprecipitates from the control strain CAF2-1 (lane 1) and the efg1 mutant HLC67 (lane 2) were analysed by immunoblotting using anti-Efg1 antiserum. The arrow indicates the migration of Efg1. (PDF) [file pgen.1005447.s001.pdf]

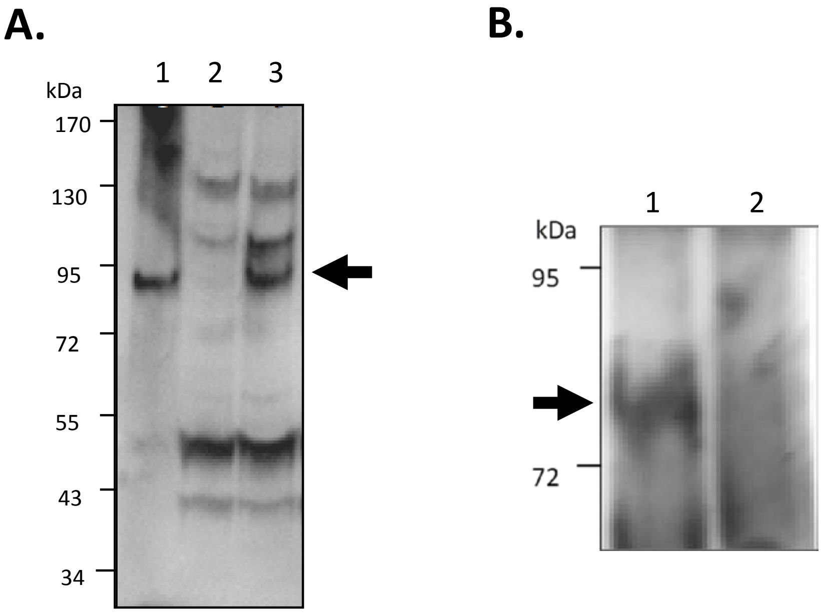

S1 Fig. Activity of rabbit anti-Efg1 antiserum. A. Immunoblotting. Extracts of *efg1* mutant HLC67 (lane 2) and control strain CAF2-1 (lane 3) were separated by SDS-PAGE and blots were probed with anti-Efg1 antiserum (1 : 5000). For comparison, 250 ng of *E. coli*-produced His_{10}-Efg1 protein was used (lane 1). **B.** Immunoprecipitation. Efg1 was immunoprecipitated using anti-Efg1 antiserum and protein G-coated agarose beads. Immunoprecipitates from the control strain CAF2-1 (lane 1) and the *efg1* mutant HLC67 (lane 2) were analysed by immunoblotting using anti-Efg1 antiserum. The arrow indicates the migration of Efg1.
