# Supplementary material for: Hypoxia and Temperature Regulated Morphogenesis in Candida albicans
Source: PLoS Genet. 2015 Aug 14;11(8):e1005447. doi: 10.1371/journal.pgen.1005447 (PMC4537295; doi:10.1371/journal.pgen.1005447)
Supplement: S2 Fig — For Efg1, genomic binding sites were derived from ChIP chip experiments comparing strains CAF2-1 (EFG1/EFG1) and HLC52 (efg1/efg1); for HA-Efg1, strains HLCEEFG1 (efg1/efg1 [HA-EFG1]) and CAF2-1 were compared. Normoxic binding sites for HA-Efg1 were obtained from Lassak et al. [40]. The shaded circle encompasses genes in filamentous growth. (PDF) [file pgen.1005447.s002.pdf]

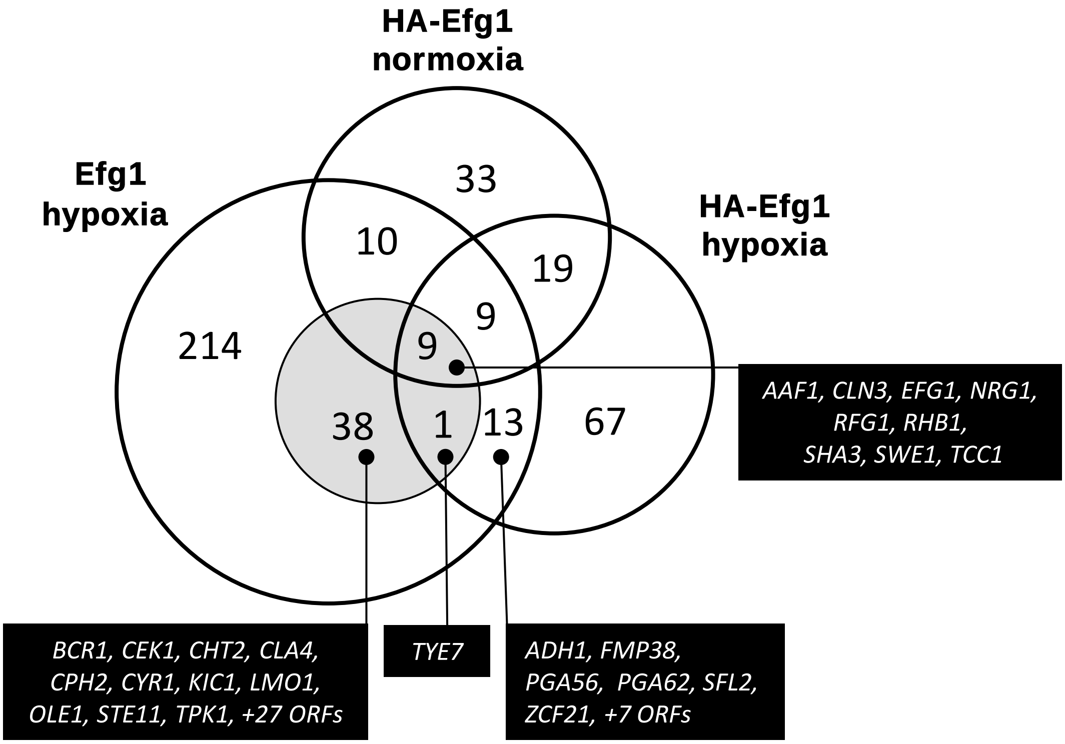

S2 Fig. Intersection of genomic binding sites for Efg1 and HA-Efg1 under hypoxia and normoxia. For Efg1, genomic binding sites were derived from ChIP chip experiments comparing strains CAF2-1 (*EFG1/EFG1*) and HLC52 (*efg1/efg1*); for HA-Efg1, strains HLCEEFG1 (*efg1/efg1* [*HA-EFG1*]) and CAF2-1 were compared. Normoxic binding sites for HA-Efg1 were obtained from Lassak *et al.* [40]. The shaded circle encompasses genes in filamentous growth.
