# Supplementary material for: Hypoxia and Temperature Regulated Morphogenesis in Candida albicans
Source: PLoS Genet. 2015 Aug 14;11(8):e1005447. doi: 10.1371/journal.pgen.1005447 (PMC4537295; doi:10.1371/journal.pgen.1005447)
Supplement: S3 Fig — GO terms for Efg1 binding targets were identified in ChIP chip data using the CGD GO Term Finder tool (http://www.candidagenome.org/cgi-bin/GO/goTermFinder); the analysis was conducted in June 2013. Genome frequencies of genes corresponding to GO terms are expressed as percentages (gene number relative to 6,525 genes in the C. albicans genome; the frequency of genes binding Efg1 that correspond to a specific GO term are expressed relative to the total number of 106 genes binding HA-Efg1). Superscripts: a, Efg1 binding in yeast normoxia [40]; b, Efg1 binding in hyphae inducing conditions [40]; c, Efg1 binding in biofilm inducing conditions [42]. P values for overrepresented categories were calculated using a hyper geometric distribution with multiple hypothesis correction according to the GO Term Finder tool website (http://www.candidagenome.org/help/goTermFinder.shtml). The P value cutoff used was 0.05. (PDF) [file pgen.1005447.s003.pdf]

GO term[1] (genome frequency)	HA-Efg1 binding genes annotated to the term[2]		
	Name	Frequency[3]	*P* value[4]
**A. Process**			
Regulation of multi-organism process (1.6 %)	*AHR1 ALS1 BMH1 BRG1 CBK1 CRZ2*[a] *CSR1 CZF1*[a] *DEF1*[a] *EFG1*[ac] *SHA3*[a] *TEC1*[a] *TYE7*	11.4 %	1.39e-05
Regulation of filamentous growth (2.6 %)	*AHR1 BMH1 BRG1 CBK1 CLN3 CZF1 DEF1 EFG1*[ac] *NRG1*[ac] *RFG1*[ac] *RME1 TEC1*[a] *TYE7 WOR2*[a]	12.3 %	0.00066
Regulation of biosynthetic process (10.9 %)	*AAF1*[ac] *ADAEC AHR1 BMH1 BRG1 CBF1*[a] *CBK1 CLN3 CRZ2*[a] *CSR1 CUP9 CZF1 EFG1*[ac] *GZF3 HTB1 NRG1*[ac] *RFG1*[ac] *RME1 RPS23A RTF1 SBP1 SFU1 SHA3*[a] *TCC1*[ac] *TEC1*[a] *TYE7, WOR2*[a] *ZCF21 ORF19.4375*	25.4 %	0.00532
***B*. Function**			
Nucleic acid binding transcription factor activity (3.4 %)	*AHR1 BRG1 CBF1*[a] *CRZ2*[a] *CSR1 CUP9 CZF1 EFG1*[ac] *GZF3 NRG1*[ac] *RFG1*[ac] *RME1 SFU1 TEC1*[a] *TYE7 WOR2 ZCF21*	14 %	3.25e-05
Sequence-specific DNA binding (2.9 %)	*AHR1 BMH1 BRG1 CBF1*[a] *CSR1 CUP9 EFG1*[ac] *GZF3 NRG1*[ac] *RFG1*[ac] *RME1 SBP1 SFU1 TEC1*[a] *TYE7 WOR3*	14.9 %	1.83e-05

S3 Fig. GO categories of genes binding HA-Efg1 under hypoxia. GO terms for Efg1 binding targets were identified in ChIP chip data using the CGD GO Term Finder tool (http://www.candidagenome.org/cgi-bin/GO/goTermFinder); the analysis was conducted in June 2013. Genome frequencies of genes corresponding to GO terms are expressed as percentages (gene number relative to 6,525 genes in the *C. albicans* genome; the frequency of genes binding Efg1 that correspond to a specific GO term are expressed relative to the total number of 106 genes binding HA-Efg1). Superscripts: a, Efg1 binding in yeast normoxia [40]; b, Efg1 binding in hyphae inducing conditions [40]; c, Efg1 binding in biofilm inducing conditions [42]. *P* values for overrepresented categories were calculated using a hyper geometric distribution with multiple hypothesis correction according to the GO Term Finder tool website (http://www.candidagenome.org/help/goTermFinder.shtml). The *P* value cutoff used was 0.05.
