# Supplementary material for: Hypoxia and Temperature Regulated Morphogenesis in Candida albicans
Source: PLoS Genet. 2015 Aug 14;11(8):e1005447. doi: 10.1371/journal.pgen.1005447 (PMC4537295; doi:10.1371/journal.pgen.1005447)
Supplement: S4 Fig — To verify the functionality of C-terminal HA-tagged Ace2 the phenotypes of three isolates of strain CLvW004 (ACE2-HA/ace2) was compared to mutant strain MK106 (ace2/ace2), wild-type strain BWP17 (ACE2/ACE2) and the heterozygous strain CLvW001 (ACE2/ace2). (A) Drop dilution assay for sensitivity to 4 μM Pmt1-inhibitor [49] and resistance to antimycin A (20 μg/ml). The agar plates were photographed after 2 d incubation at 30°C. The ace2 mutant strain Mk106 shows enhanced sensitivity to the Pmt1-inhibitor and is less susceptible to the respiratory inhibitor antimycin A [30]. (B) Colonies of the indicated strains were photographed following growth for 2 d at 30°C on YPD agar. The ace2 mutant strain Mk106 shows the wrinkled colony phenotype described previously [24]. Phenotypes of strains CLvW004.1–3 correspond to the heterozygous strain CLvW001 indicating that the Ace2-HA protein in these strains is functional. (PDF) [file pgen.1005447.s004.pdf]

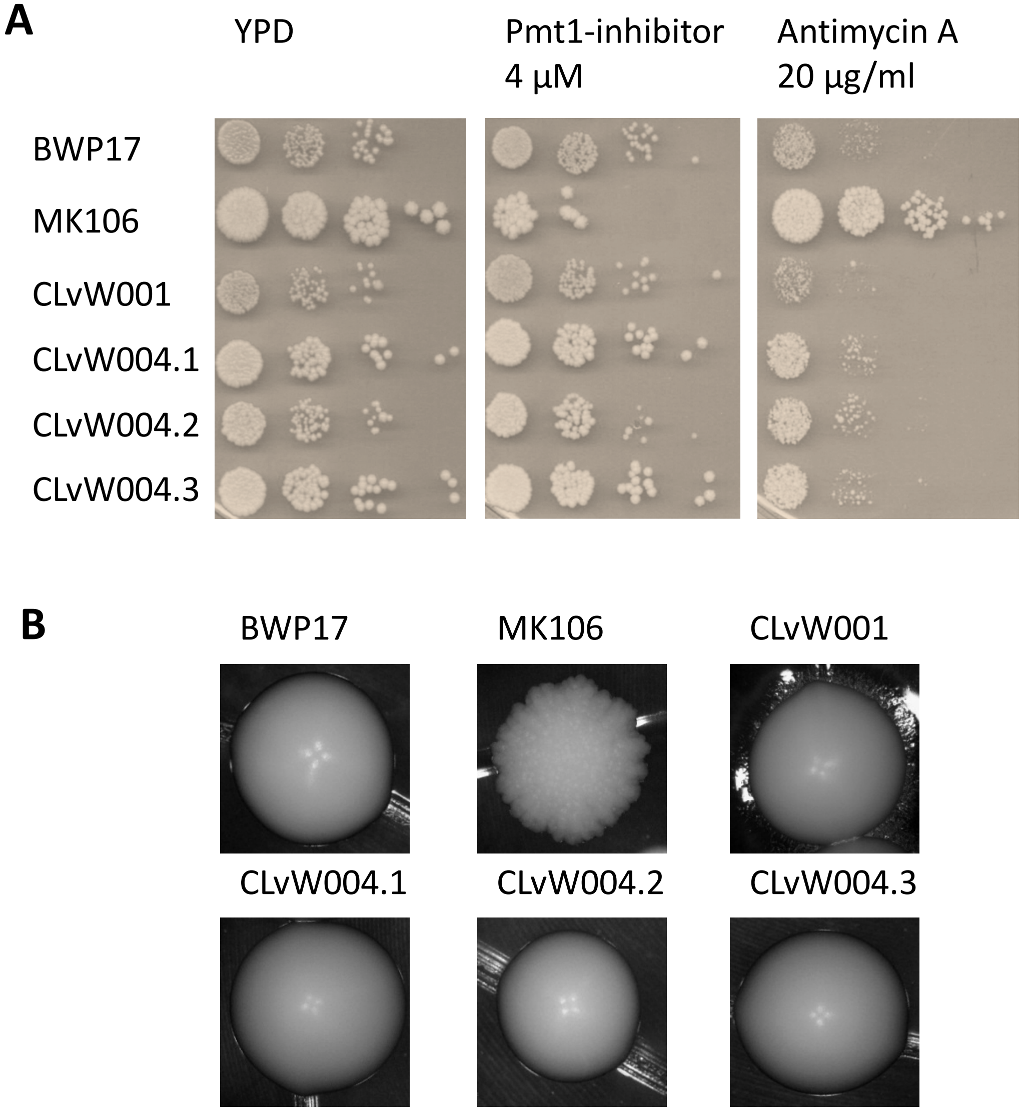

S4 Fig. Functionality of the HA tagged Ace2 protein. To verify the functionality of C-terminal HA-tagged Ace2 the phenotypes of three isolates of strain CLvW004 (*ACE2-HA*/*ace2*) was compared to mutant strain MK106 (*ace2*/*ace2*), wild-type strain BWP17 (*ACE2*/*ACE2*) and the heterozygous strain CLvW001 (*ACE2*/*ace2*). (A) Drop dilution assay for sensitivity to 4 µM Pmt1-inhibitor and resistance to antimycin A (20 µg/ml). The agar plates were photographed after 2 d incubation at 30 °C. The *ace2* mutant strain Mk106 shows enhanced sensitivity to the Pmt1-inhibitor [49] and is less susceptible to the respiratory inhibitor antimycin A [30]. (B) Colonies of the indicated strains were photographed following growth for 2 d at 30 °C on YPD agar. The *ace2* mutant strain Mk106 shows the wrinkled colony phenotype described previously [27]. Phenotypes of strains CLvW004.1-3 correspond to the heterozygous strain CLvW001 indicating that the Ace2-HA protein is functional.
