# Supplementary material for: Hypoxia and Temperature Regulated Morphogenesis in Candida albicans
Source: PLoS Genet. 2015 Aug 14;11(8):e1005447. doi: 10.1371/journal.pgen.1005447 (PMC4537295; doi:10.1371/journal.pgen.1005447)
Supplement: S5 Fig — Venn diagram showing numbers of genes bound by HA-tagged Ace2 under normoxic conditions (30°C, YPD) and hypoxic conditions (30°C, YPD 0.2% O2 and 6% CO2). Binding regions with the corresponding ORFs are listed in S2 and S3 Table. Genomic binding sites were derived from ChIP chip experiments comparing strains ClvW004 (ACE2-HA/ace2) and BWP17 (non tag control). (PDF) [file pgen.1005447.s005.pdf]

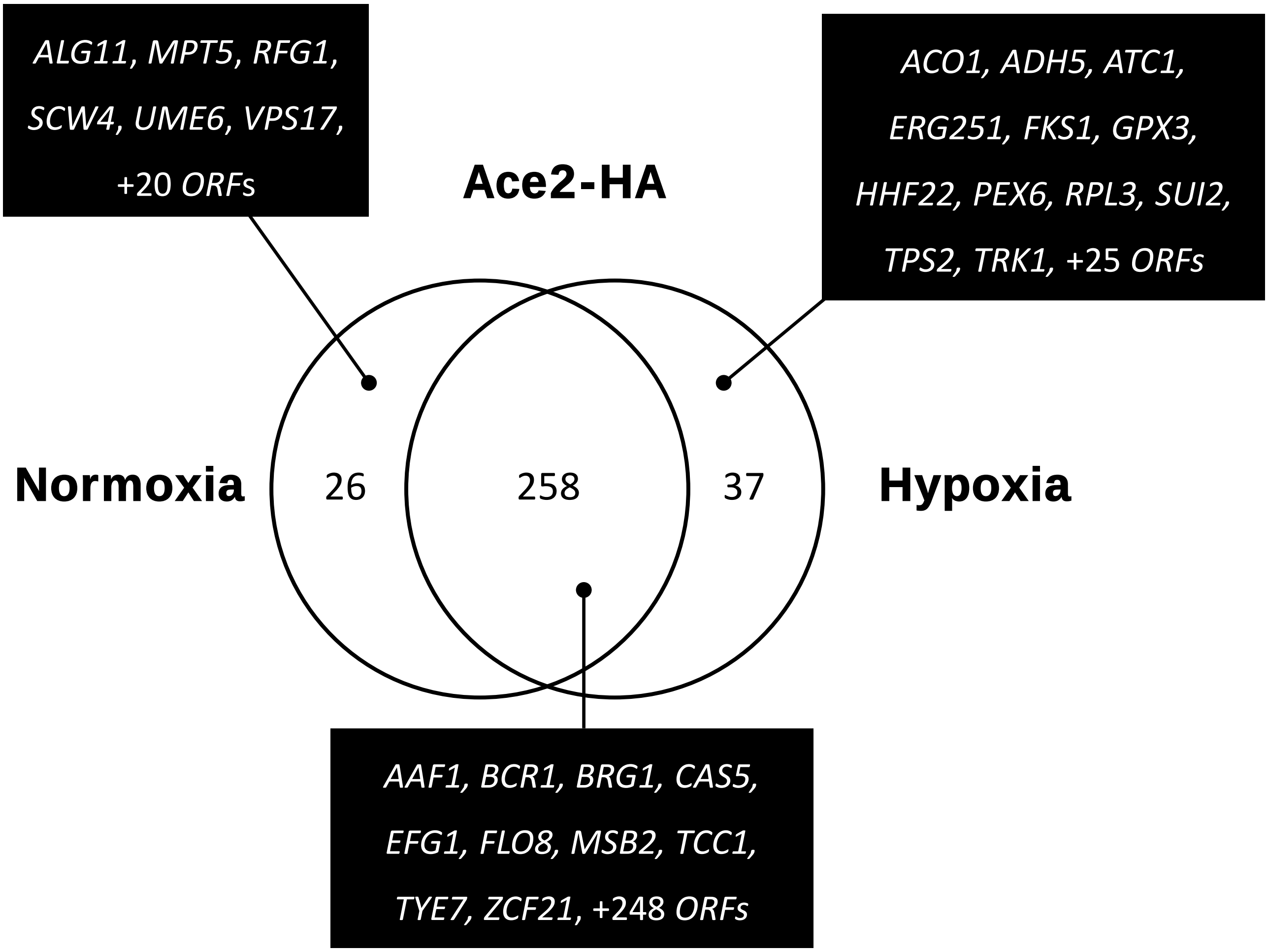

S5 Fig. **Genomic binding sites for Ace2**. Venn diagram showing numbers of genes bound by HA-tagged Ace2 under normoxic conditions (30 °C, YPD) and hypoxic conditions (30 °C, YPD 0.2 % O_2 and 6 % CO_2). Binding regions with the corresponding ORFs are listed in supplementary table S2 and S3. Genomic binding sites were derived from ChIP chip experiments comparing strains CLvW004 (*ACE2-HA/ace2*) and BWP17 (non tag control). For both conditions, 258 genes are common.
