# Supplementary material for: Hypoxia and Temperature Regulated Morphogenesis in Candida albicans
Source: PLoS Genet. 2015 Aug 14;11(8):e1005447. doi: 10.1371/journal.pgen.1005447 (PMC4537295; doi:10.1371/journal.pgen.1005447)
Supplement: S6 Fig — (A) Binding positions identified by ChIP chip experiments. Binding positions of Efg1 (black ovals), Ace2-HA (orange ovals) and HA-Efg1 positions (grey ovals) identified by ChIP chip experiments are shown schematically. (B) Quantitation of Ace2, HA-Efg1 and Efg1 enrichment by ChIP on target promoters. Following ChIP the respective fold enrichment was determined by qPCR using oligonucleotide pairs shown by red arrows in A. Chromatin of strains CAF2–1 (Efg1) and HLCEEFG1 (HA-Efg1) was immunoprecipitated using anti-Efg1 and anti-HA antibody, respectively, as described in S1 Table. For Ace2 enrichment anti-HA antibody was used for immunoprecipitation of strain CLvW004 (Ace2-HA), as described in S2 Table. In a control experiment, ChIP followed by qPCR was also done using anti-Efg1 antibody on extracts of strain HLCEEFG1 (HA-Efg1). qPCR experiments were done using two biological replicates, which were assayed in triplicate. The mean fold enrichment (± standard deviation) for each protein was calculated relative to the respective no tag or mutant strain and normalized to the input sample. Statistical relevance is indicated by asterisks: *, P < 0.05; **, P < 0.01; ***, P < 0.001. The results verify the presence of proteins on target promoters; in addition, the presence of HA-Efg1 on the BCR1 promoter, which was not found in the ChIP chip experiment, was revealed by the ChIP-qPCR experiment. (PDF) [file pgen.1005447.s006.pdf]

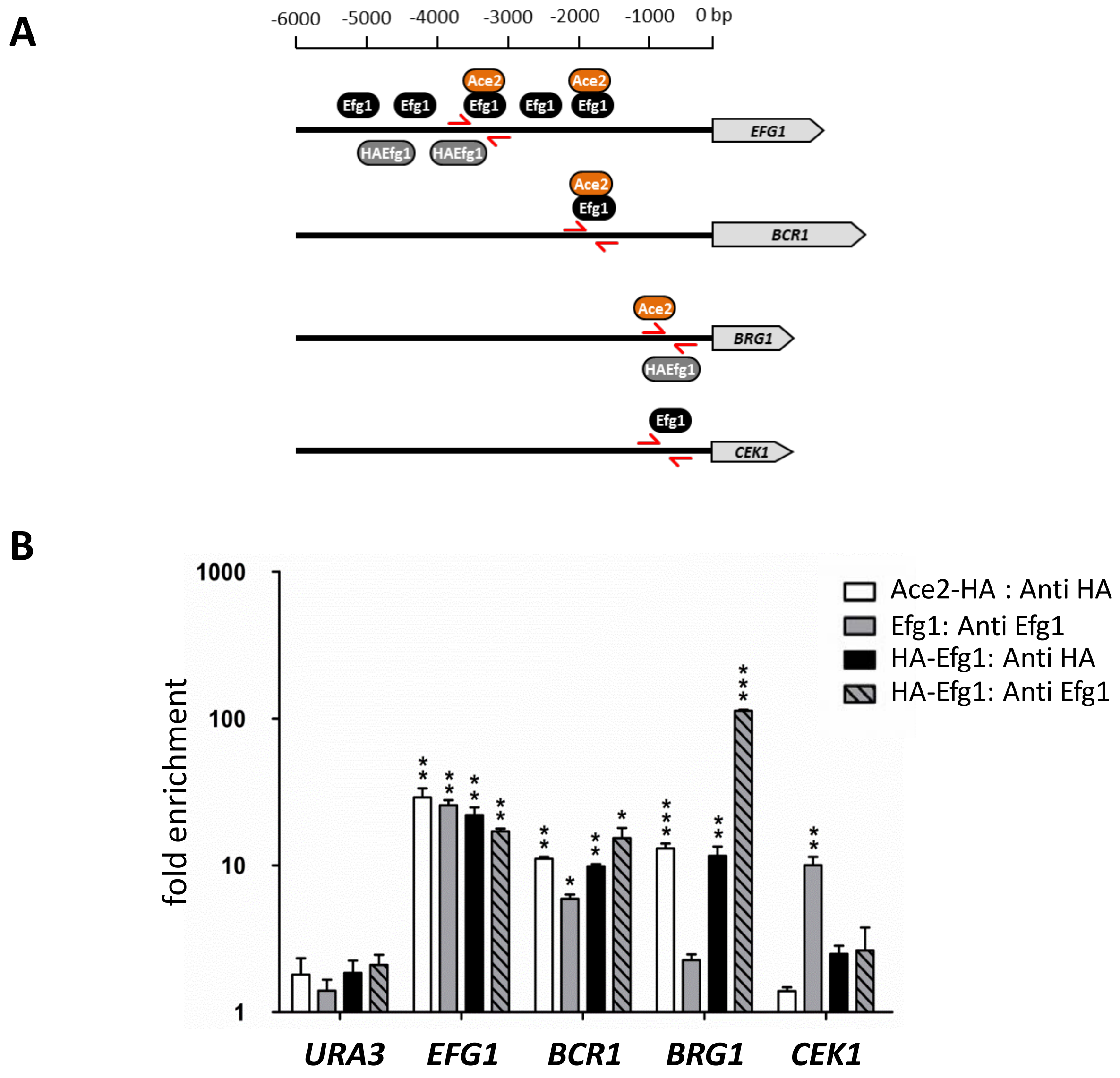

S6 Fig. Genomic localization of Ace2 and Efg1 on target promoters. (A) Binding positions identified by ChIP chip experiments. Binding positions of Efg1 (black ovals), Ace2-HA (orange ovals) and HA-Efg1 positions (grey ovals) identified by ChIP chip experiments are shown schematically. (B) Quantitation of Ace2, HA-Efg1 and Efg1 enrichment by ChIP on target promoters. Following ChIP the respective fold enrichment was determined by qPCR using oligonucleotide pairs shown by red arrows in A. Chromatin of strains CAF2 -1 (Efg1) and HLCEEFG1 (HA-Efg1) was immunoprecipitated using anti-Efg1 and anti-HA antibody, respectively, as described in S1 Table. For Ace2 enrichment anti-HA antibody was used for immunoprecipitation of strain CLvW004 (Ace2-HA), as described in S2 Table. In a control experiment, ChIP followed by qPCR was also done using anti-Efg1 antibody on extracts of strain HLCEEFG1 (HA-Efg1). qPCR experiments were done using two biological replicates, which were assayed in triplicate. The mean fold enrichment (± standard deviation) for each protein was calculated relative to the respective no tag or mutant strain and normalized to the input sample. Statistical relevance is indicated by asterisks: *, $P < 0.05$; **, $P < 0.01$; ***, $P < 0.001$. The results verify the presence of proteins on target promoters; in addition, the presence of HA-Efg1 on the *BCR1* promoter, which was not found in the ChIP chip experiment, was revealed by the ChIP-qPCR experiment.
