# Supplementary material for: Hypoxia and Temperature Regulated Morphogenesis in Candida albicans
Source: PLoS Genet. 2015 Aug 14;11(8):e1005447. doi: 10.1371/journal.pgen.1005447 (PMC4537295; doi:10.1371/journal.pgen.1005447)
Supplement: S7 Fig — Strains CAF2-1 (control), the ace2 mutant MK106 and the efg1 mutant HLC52 were precultured under normoxia at 30°C in YPD medium and used for inoculation of 100 ml YPD cultures. Cultures were incubated at 30°C under normoxia, or normoxia with addition of CO2 (6% CO2), or under hypoxia (0.2% O2), or under hypoxia with addition of CO2 (0.2% O2, 6% CO2) until OD600 = 0.5 and total RNA was then isolated. Relative transcript levels were determined using ACT1 transcript as the reference as described in Fig 4. Relative transcript levels (RTL) for the AAF1, TYE7 and ZCF21 transcripts in strains CAF2-1 (control), HLC52 (efg1) and MK106 (ace2). Error bars represent standard deviation of the means. A two-tailed, unpaired t test comparing the cycle threshold values of samples grown in hypoxic and normoxic conditions for each mutant respectively was used to determine the statistical relevance: *, P < 0.05; **, P < 0.01; ***, P < 0.001. (PDF) [file pgen.1005447.s007.pdf]

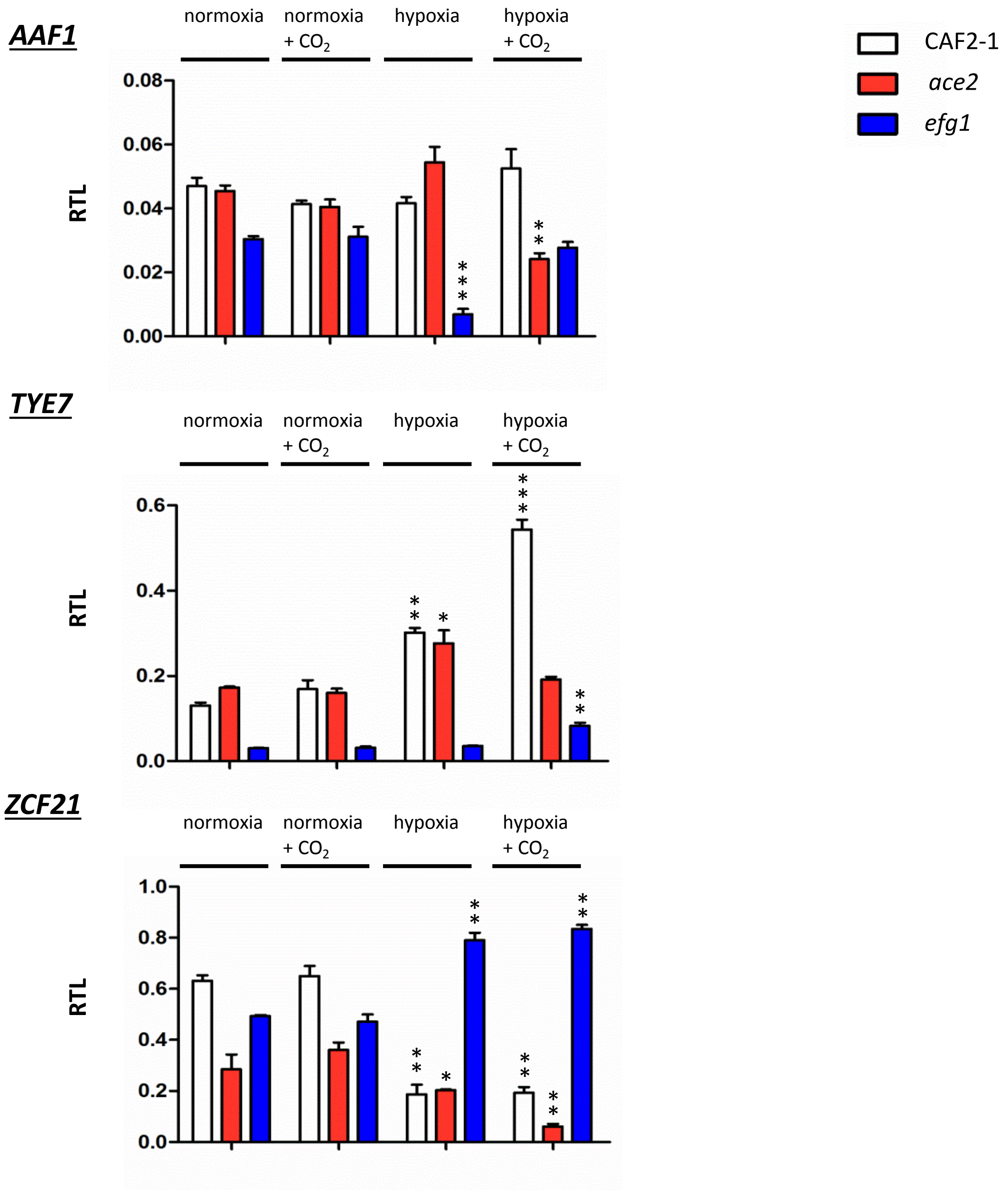

S7 Fig. Transcriptional regulation of selected Efg1 and Ace2 target genes under hypoxia. Strains CAF2-1 (control), the *ace2* mutant MK106 and the *efg1* mutant HLC52 were precultured under normoxia at 30 °C in YPD medium and used for inoculation of 100 ml YPD cultures under normoxia, or normoxia with addition of CO_2 (6 % CO_2), or under hypoxia (0.2 % O_2), or under hypoxia with addition of CO_2 (0.2 % O_2, 6 % CO_2). Cultures were incubated at 30 °C in the respective condition until OD_{600}= 0.5 and total RNA was then isolated. Relative transcript levels were determined using *ACT1* transcript as the reference as described in Fig. 4. Relative transcript levels (RTL) for the *AAF1*, *TYE7* and *ZCF21* transcripts in strains CAF2-1 (control), HLC52 (*efg1*) and MK106 (*ace2*). Error bars represent standard deviation of the means. A two-tailed, unpaired *t* test comparing the cycle threshold values of samples grown in hypoxic and normoxic conditions for each mutant respectively was used to determine the statistical relevance: *, $P < 0.05$; **, $P < 0.01$; ***, $P < 0.001$.
