# Supplementary material for: Hypoxia and Temperature Regulated Morphogenesis in Candida albicans
Source: PLoS Genet. 2015 Aug 14;11(8):e1005447. doi: 10.1371/journal.pgen.1005447 (PMC4537295; doi:10.1371/journal.pgen.1005447)
Supplement: S8 Fig — The strains were grown under normoxia without or with 6% CO2 on YPS agar for 4 d at 25°C or at 37°C for 3 d. Strains included CAF2-1 (control), homozygous single mutants HLC52 (efg1), CJN702 (bcr1), MK106 (ace2), TF022 (brg1) and double knockout strains PDEB4 (efg1 brg1), PDBB4 (bcr1 brg1) and CLvW024 (bcr1 ace2). ACE2 could not be disrupted in an efg1 mutant background; therefore, the heterozygous mutant strain CLvW047 (efg1/EFG1, ace2/ace2) was constructed and its phenotype was compared to the efg1/EFG1 strain DSC11. (PDF) [file pgen.1005447.s008.pdf]

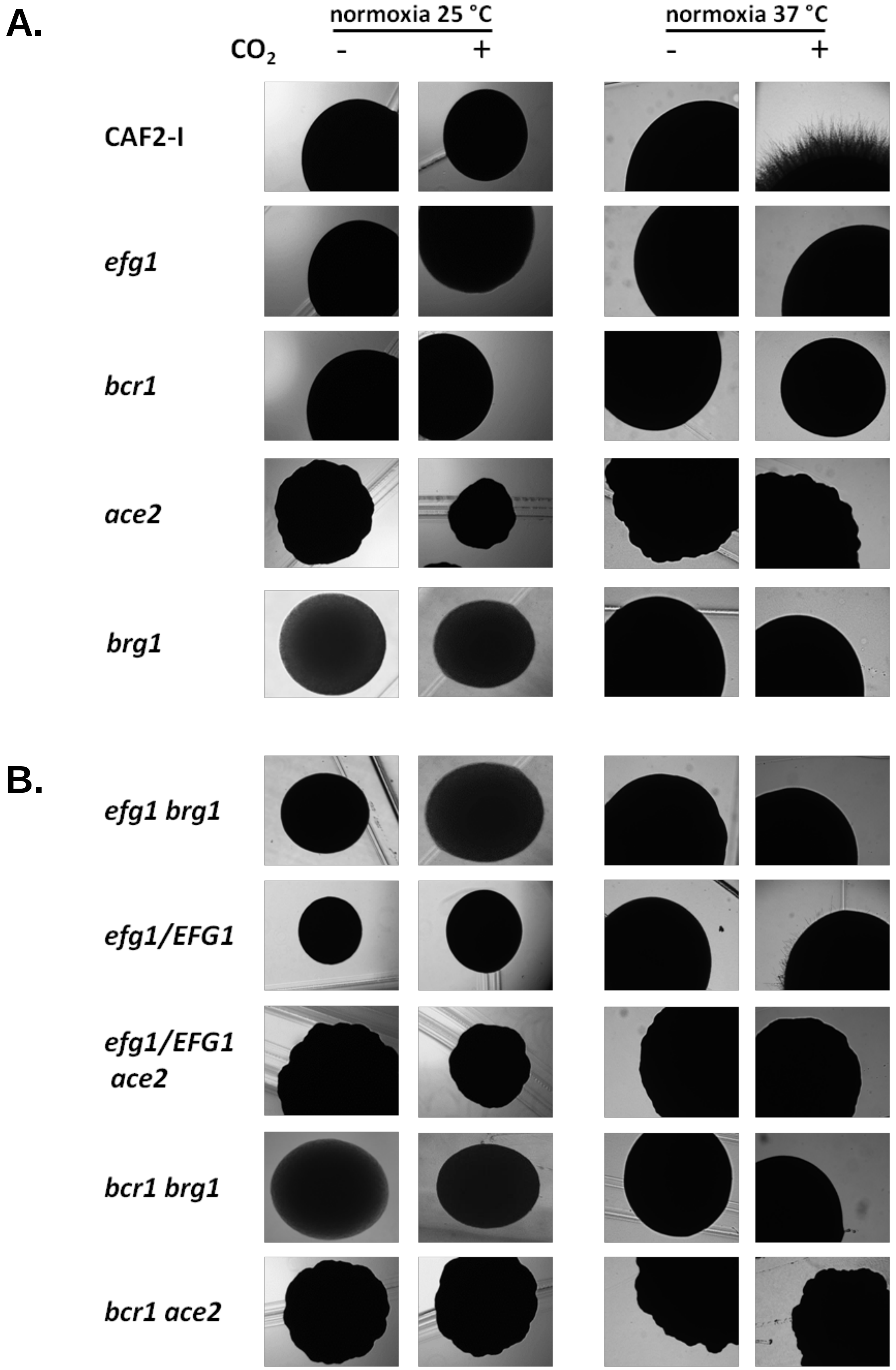

S8 Fig. Normoxic phenotypes of mutants lacking hypoxic regulators under hypoxia. The strains were grown under normoxia without or with 6 % CO_2 on YPS agar for 4 d at 25 °C or for 3d at 37 °C. Strains included CAF2-1 (control), homozygous single mutants HLC52 (*efg1*), CJN702 (*bcr1*), MK106 (*ace2*), TF022 (*brg1*) and double knockout strains PDEB4 (*efg1 brg1*), PDBB4 (*bcr1 brg1*) and CLvW024 (*bcr1 ace2). ACE2* could not be disrupted in an *efg1* mutant background; therefore, the heterozygous mutant strain CLvW047 (*efg1/EFG1, ace2*/*ace2*) was constructed and its phenotype was compared to the *efg1/EFG1* strain DSC11.
