# Supplementary material for: Hypoxia and Temperature Regulated Morphogenesis in Candida albicans
Source: PLoS Genet. 2015 Aug 14;11(8):e1005447. doi: 10.1371/journal.pgen.1005447 (PMC4537295; doi:10.1371/journal.pgen.1005447)
Supplement: S4 Table — (PDF) [file pgen.1005447.s012.pdf]

S4 Table. ***C. albicans*** **strains**

**Strain**	**Genotype**	**reference/source**
SC5314	wild-type	[1]
CAF2-1	*URA3/ura3*::*imm434*	[1]
CAI4	*ura3*::*imm434/ura3*::*imm434*	[1]
HLC52	Like CAI4 but *efg1*::*hisG/efg1*::*hisG-URA3-hisG*	[2]
HLC67	Like CAI4 but *efg1*::*hisG/efg1*::*hisG*	[2]
HLC46	Like CAI4 but *EFG1/efg1*::*hisG-URA3-hisG*	[2]
BCA0901	Like CAI4, but *EFG1/efg1*::*hisG-URA3-hisG*	[3]
JKC19	Like CAI4, but *cph1*::*hisG/cph1*::*hisG-URA3-hisG*	[4]
HLC54	Like CAI4 but *cph1*::*hisG/cph1*::*hisG efg1/efg1*::*hisG-URA3-hisG*	[2]
HLCE	Like HLC67 but *efg1*::*hisG/efg1*:: [*EFG1p-URA3*]	[5]
HLCEEFG1	Like HLC67, but *efg1*::*hisG/efg1*::[*EFG1p-HA-EFG1-URA3*] (pTD38 HA/Pac1 integrated in *EFG1p*)	[5]
HLCPEFG1	Like HLC67, but *efg1*::*hisG/efg1*::[*EFG1p-EFG1-URA3*] (pPRDA208C/Pac1 integrated in *EFG1p*)	this study
HLCNEFG1	Like HLC67, but *efg1*::*hisG/efg1*::[*EFG1p-ΔN-EFG1-URA3*] (pPDNEFG1/Pac1 integrated in *EFG1p*)	this study
HLC67[pDB1]	Like HLC67, but *LEU2/leu2*::*PCK1p-EFG1*^{T206A}*-URA3*	[6]
HLC67[pDB2]	Like HLC67, but *LEU2/leu2*::*PCK1p-EFG1*^{T206E}*-URA3*	[6]
HLCEEFG1T206A	Like HLC67, but *efg1*::*hisG/efg1*::[*EFG1p-EFG1*^{T206A}*-URA3*] (pPDEFG1T206A/Pac1 integrated in *EFG1p*)	this study
HLCEEFG1T206E	Like HLC67, but *efg1*::*hisG/efg1*::[*EFG1p-EFG1*^{T206E}*-URA3*] (pPDEFG1T206E/Pac1 integrated in *EFG1p*)	this study
HLCEEFG1T179A	Like HLC67, but *efg1*::*hisG/efg1*::[*EFG1p-EFG1*^{T179A}*-URA3*] (pPDEFG1T179A/Pac1 integrated in *EFG1p*)	this study
HLCEEFG1T179E	Like HLC67, but *efg1*::*hisG/efg1*::[*EFG1p-EFG1*^{T179E}*-URA3*] (pPDEFG1T179E/Pac1 integrated in *EFG1p*)	this study

CEC2907	Like CAI4, but *his1*::*hisG/HIS1 arg4*::*hisG/ARG4 ADH1/adh1*::*TDH3p-carTA*::*SAT1*	[7]
CECSTE11	Like CEC2907, but *RPS1/RPS1*::*TETp-STE11-URA3*<br>(pClp10TETSTE11/StuI integrated in *RPS1* locus)	this study
CECCEK1	Like CEC2907, but *RPS1/RPS1*::*TETp-CEK1-URA3*<br>(pClp10TETCEK1/StuI integrated in *RPS1* locus)	this study
CECCPH1	Like CEC2907, but *RPS1/RPS1*::*TETp-CPH1-URA3*<br>(pClp10TETCPH1/StuI integrated in *RPS1* locus)	this study
DSC11	*efg1::hisG/efg1::hisG::EFG1-dpl200 ura3*::*imm434/ura3*::*imm434::URA3*	[8]
BWP17	*ura3*::*imm434/ura3*::*imm434, his1*::*hisG/his1*::*hisG arg4*::*hisG/arg4*::*hisG*	[9]
MK106	Like SC5314, but *ace2*::*FRT/ace2::FRT*	[10]
CLvW001	Like BWP17, but *ace2*::*lacZ-SAT1/ACE2*	this study
CLvW004	Like CLvW001, but *ace2*::*SAT1/ACE2*^{HA}::*URA3*	this study
CLvW008	Like CAI4, but *ace2*::*FRT/ace2*::*FRT*	this study
SN250	*ura3*Δ::*imm434*::*URA3-IRO1/ura3*Δ::*imm434 arg4*::*hisG/arg4::hisG his1*::*hisG/his1*::*hisG*<br>*leu2*::*hisG*::*CdHIS1/leu2*::*hisG*::*CmLEU2*	[11]
CLvW047	Like CLvW008, but *efg1*::*hisG-URA3-hisG/EFG1*	this study
CJN702	Like Bwp17, but *his1*::*hisG/his1*::*hisG*::*pHIS1 bcr1*::*URA3/bcr1*::*ARG4*	[12]
CLvW024	Like CJN702, but *ace2*::*FRT/ace2*::*FRT*	this study
TF022	*ura3Δ::imm434::URA3-IRO1/ura3*Δ::*imm434 arg4*::*hisG/arg4*::*hisG his1*::*hisG/his1*::*hisG*<br>*leu2*::*hisG/leu2*::*hisG brg1*Δ::*CmLEU2/brg1*Δ::*CdHIS1*	[13]
PDEB4	Like HLC52, but *brg1*::*FRT/brg1*::*FRT*	this study
PDBB4	Like CJN702, but *brg1*::*FRT/brg1*::*FRT*	this study

References

1. Fonzi WA, Irwin MY. Isogenic strain construction and gene mapping in *Candida albicans*. Genetics. 1993;134: 717-728.

2. Lo HJ, Köhler JR, DiDomenico B, Loebenberg D, Cacciapuoti A, Fink GR. Nonfilamentous *C. albicans* mutants are avirulent. Cell. 1997;90: 939-949.
3. Braun BR, Johnson AD. *TUP1*, *CPH1* and *EFG1* make independent contributions to filamentation in *Candida albicans*. Genetics. 2000;155: 57-67.
4. Liu H, Köhler J, Fink GR. Suppression of hyphal formation *in Candida albicans* by mutation of a STE12 homolog. Science. 1994;266: 1723-1726. Erratum in Science. 1995;267:217.
5. Noffz CS, Liedschulte V, Lengeler K, Ernst JF. Functional mapping of the *Candida albicans* Efg1 regulator. Eukaryot Cell. 2008;7: 881-893.
6. Bockmühl DP, Ernst JF. A potential phosphorylation site for an A-type kinase in the Efg1 regulator protein contributes to hyphal morphogenesis of *Candida albicans*. Genetics. 2001;157: 1523-30.
7. Chauvel M, Nesseir A, Cabral V, Znaidi S, Goyard S, Bachellier-Bassi S, et al. A versatile overexpression strategy in the pathogenic yeast *Candida albicans*: identification of regulators of morphogenesis and fitness. PLoS One. 2012;7: e45912.
8. Park H, Myers CL, Sheppard DC, Phan QT, Sanchez AA, Edwards JE, Filler SG. Role of the fungal Ras-protein kinase A pathway in governing epithelial cell interactions during oropharyngeal candidiasis. Cell Microbiol 2005;7: 499–510.
9. Wilson RB, Davis D, Mitchell AP. Rapid hypothesis testing with *Candida albicans* through gene disruption with short homology regions. J Bacteriol. 1999;181: 1868-1874.

10. Kelly MT, MacCallum DM, Clancy SD, Odds FC, Brown AJ, Butler G. The *Candida albicans CaACE*2 gene affects morphogenesis, adherence and virulence. Mol Microbiol. 2004;53: 969-983.

11. Noble SM, French S, Kohn LA, Chen V, Johnson AD. Systematic screens of a *Candida albicans* homozygous deletion library decouple morphogenetic switching and pathogenicity. Nat Genet. 2010;42: 590-598.

12. Nobile CJ, Mitchell AP. Regulation of cell-surface genes and biofilm formation by the *C. albicans* transcription factor Bcr1p. Curr Biol. 2005;15: 1150-1155.

13. Homann OR, Dea J, Noble SM, Johnson AD. A phenotypic profile of the *Candida albicans* regulatory network. PLoS Genet. 2009;5: e1000783.
