# Supplementary material for: Hypoxia and Temperature Regulated Morphogenesis in Candida albicans
Source: PLoS Genet. 2015 Aug 14;11(8):e1005447. doi: 10.1371/journal.pgen.1005447 (PMC4537295; doi:10.1371/journal.pgen.1005447)
Supplement: S5 Table — (PDF) [file pgen.1005447.s013.pdf]

S5 Table. List of oligonucleotides

name	sequence
pET19Serinhin	5’-GTATGGGTATCAATCGAATTATTACCAGGG-3’
pET19Serinher	5’-CCCTGGTAATAATTCGATTGATACCCATAC-3’
EFG1BamHIFor	5’-ACTACGCGGATCCATGTCAACGTATTCTATACCC -3’
EFG1BamHIRev	5’-GGTATGCGGATCCCTTTTCTTCTTTGGCAACAG-3’
Efg1179AFor	5’GTATCGACAATGCAACAACCAGCTCCTGTTCAGGATACGTTGAAC-3’
Efg1179ARev	5’ GTTCAACGTATCCTGAACAGGAGCTGGTTGTTGCATTGTCGATAC-3’
UTREFG1For	5’-GCGTTAGTCTCAATTGAACAGATAG-3’
EFG1seqFor	5’-GACAACCAGGTCAACAGACTG-3’
EFG1seqRev	5’-CAGTGGCAGCCTTGGTATTTAC-3’
EFG1seqM	5’-CAATTCTACCAGTGGACCTTC-3’
CIp UL	5’ -ATACTACTGAAATTTCCTGACTTTC-3’
CIpUR	5’ –ATTACTATTTACAATCAAAGGTGGTC-3’
lacZACE2For	5’-ACTGAAATGCAATCTTCTCTCCCACTCGAGCAAACACTCTCTCCATG AAAAACCAACTTGTGGCAGAAGTCTCGACATTTTATGATGGAATG-3’
lacZACE2Rev	5’-ATTTAGTGAAGGAGGTGCAAAAGTTGGGAAAGTAAATGA GAAAAAGAAAAAATGGTAAATAAAATACTTGAAAGGTATTTCATGAAC ATGACTGAAAAAATTC-3’
ACE2HAFor	5’-CAGTGGTTGAGAGGATAGAAAAACAGTTGCTACAGGAAG ATAAGAGTGTTACTGAGGAGTTTTTAATGTTGCAATCGGGTGGTGGTC GGATCCCCGGGTTAATTAAC-3’
ACE2HARev	5’-GCAATCTCTAACAGAAGTGAACATAGAATTAATAAACTG GCAGTAAATCAAACGATATAAAATAAAAAAGAATAATAGCTAGAAGG ACCACCTTTGATTG-3’
ACE2For	5’-GATTAATGGATCTCCCGATTGG-3’
HARev	5’-CTAGAAGGACCACCTTTGATTG 3’
CAF1ApaI	5’-TATATGGTGTAAATCTCACTG-3’
CAF2XhoI	5’-TTTCCAATGCATCTCGAGTGAAGG-3’
CAR1SacII	5’-AGACTTCTGCCGCGGGTTGGTTTT-3’
CAR2SacI	5’-ATTTGTTGGGAGCTCCACAACACT-3’
CAF3ApaI	5’-TTTCGAAAGGGCCCTCTTTCTTT-3’
CAF4XhoI	5’-ATCATTGGCCTCGAGACTTAAAAA-3’
CAR3SacII	5’-GCAAATGTCCGCGGGGTAAAGA-3’
CAR4SacI	5’-CAACATTAAGAGCTCCTCAGTAAC-3’
FLP1	5’-TTCCGTTATGTGTAATCATCC-3’
ACE2UTR5	5’-ATATGGTAAGCTTCAGTTCG-3’
ACE2RTFor	5’-CGCCGAATCAAAAAGACTTC-3’
ACE2RTRev	5’-CGCACATTGTATCGACGAGT-3’
EFG1RTFor	5’-TAACGGAACCAAATTGCTCA-3’
EFG1RTRev	5’-CACCTCGAGGATTGACAGAA-3’
AAF1RTFor	5’-GCCCTTGGTGGTACATCTTT-3’
AAF1RTRev	5’-GATGGATGAGTGGATGTTGC-3’
TYE7RTFor	5’-GATTGCAAATTCTGTTCCGA-3’
TYE7RTRev	5’-AATAGGATTTGGTGGTTGGC-3’
BCR1RTFor	5’-AATGCCTGCAGGTTATTTGG-3’
BCR1RTRev	5’-GTTCTTGACCACCACCCATT-3’
ZCF21RTFor	5’-AGATCTATGATGGATATTTATCAGAAGG- 3’
ZCF21RTRev	5’-AGATCTTCAAGTGATCAATTTGGAAAT- 3’
ACT1RTFor	5’-TTGGATTCTGGTGATGGTGT-3’

ACT1RTRev	5’-TGGACAAATGGTTGGTCAAG-3’
STE11 RTFor	5’-GCTCATGATCGCCATTCTAC-3’
STE11 RTRev	5’-CTCAATCCGGCATAGTCAGA-3’
CPH1 RTFor	5’-AGATGCCTTGGAACGAGATT-3’
CPH1RTRev	5’-TGGAAGACTCGTCGTAATGG-3’
CEK1RTFor	5’-ACAACAACAACAGGCAGCAG-3’
CEK1RTRev	5’-TCCATATGCTCCTTCTCCAA-3’
CYR1 RTFor	5’-AAGAATCCCGAAACAGGAGA-3’
CYR1 RTRev	5’-CTTGTTCTGGCACGTTTGTT-3’
TPK1 RTFor	5’-TTGGATTTGCTAAAGAAGTTCAA-3’
TPK1 RTRev	5’-ACCAAATGACCACCAATCAA-3’
KIC1 RTFor	5’-CAACCAACCACCCACAATTA-3’
KIC1 RTRev	5’-GGTGACGTATTCAACGAGGA-3’
BRG1 RTFor	5’-AGAGGTCCAAAGGGTGTGAG-3’
BRG1 RTRev	5’-TACCTCTTCTGCTGCCAATG-3’
BRG15 UTR KpnI For	5’-CATGCGAGGTACCGCCGGAGATTCAGAGCTCTTAC-3’
BRG15UTR KpnI Rev	5’-CATTCGGGTACCAATTTGAATTTCTGGAATAGTAG-3’
BRG13UTR Not1 For	5’-CATGCTGCGGCCGCGTATTCCTGTTGCTTACCC-3’
BRG13UTR SacI Rev	5’-CATCGACGAGCTCGTCGTCTGGACGATCTGATTTGCTCTC-3’
BRG1UpFor	5’-GTAATGTGTGCACTACACAATAACC-3’
BRG1MidRev	5’-GTGTAACCCACATTAGGTGGCATACC-3’
URA3 CHIP For	5’-GGAGTTGGATTAGATGATAAAGGTGATGG-3‘
URA3 CHIP Rev	5’-GGACCACCTTTGATTGTAAATAGTAATA-3‘
EFG1 CHIP For	5’-CAACTTGGTCCAAGAATTC-3‘
EFG1 CHIP Rev	5’-TATGGGTTATATTCTTGGTAGTC-3‘
BCR1 CHIP For	5’-GTCCTCCCCATCCAACTA-3‘
BCR1 CHIP Rev	5’-CAAAAATAGGGTAGGCAATAGA-3‘
BRG1 CHIP For	5’-GCCGGAGATTCAGAGCTC-3‘
BRG1 CHIP Rev	5’-ACTGAGAACAGGCAATCTCC-3‘
CEK1 CHIP For	5’-GCATCGTCGAATATGTACAACC-3‘
CEK1 CHIP Rev	5’-GGCGGGTTAGATGATGAAAT-3‘
